# Targeting cytokine and chemokine signaling pathways for cancer therapy

**DOI:** 10.1038/s41392-024-01868-3

**Published:** 2024-07-22

**Authors:** Ming Yi, Tianye Li, Mengke Niu, Haoxiang Zhang, Yuze Wu, Kongming Wu, Zhijun Dai

**Affiliations:** 1 Department of Breast Surgery, The First Affiliated Hospital, College of Medicine, Zhejiang University, Hangzhou, 310000 People’s Republic of China; 2 Department of Gynecology, The Second Affiliated Hospital of Zhejiang University School of Medicine, Hangzhou, 310000 People’s Republic of China; 3 Department of Oncology, Tongji Hospital of Tongji Medical College, Huazhong University of Science and Technology, Wuhan, 430030 People’s Republic of China; 4 Department of Hepatopancreatobiliary Surgery, Fujian Provincial Hospital, Fuzhou, 350001 People’s Republic of China

**Keywords:** Cancer microenvironment, Cancer therapy

## Abstract

Cytokines are critical in regulating immune responses and cellular behavior, playing dual roles in both normal physiology and the pathology of diseases such as cancer. These molecules, including interleukins, interferons, tumor necrosis factors, chemokines, and growth factors like TGF-β, VEGF, and EGF, can promote or inhibit tumor growth, influence the tumor microenvironment, and impact the efficacy of cancer treatments. Recent advances in targeting these pathways have shown promising therapeutic potential, offering new strategies to modulate the immune system, inhibit tumor progression, and overcome resistance to conventional therapies. In this review, we summarized the current understanding and therapeutic implications of targeting cytokine and chemokine signaling pathways in cancer. By exploring the roles of these molecules in tumor biology and the immune response, we highlighted the development of novel therapeutic agents aimed at modulating these pathways to combat cancer. The review elaborated on the dual nature of cytokines as both promoters and suppressors of tumorigenesis, depending on the context, and discussed the challenges and opportunities this presents for therapeutic intervention. We also examined the latest advancements in targeted therapies, including monoclonal antibodies, bispecific antibodies, receptor inhibitors, fusion proteins, engineered cytokine variants, and their impact on tumor growth, metastasis, and the tumor microenvironment. Additionally, we evaluated the potential of combining these targeted therapies with other treatment modalities to overcome resistance and improve patient outcomes. Besides, we also focused on the ongoing research and clinical trials that are pivotal in advancing our understanding and application of cytokine- and chemokine-targeted therapies for cancer patients.

## Introduction

**Fig. 1:**
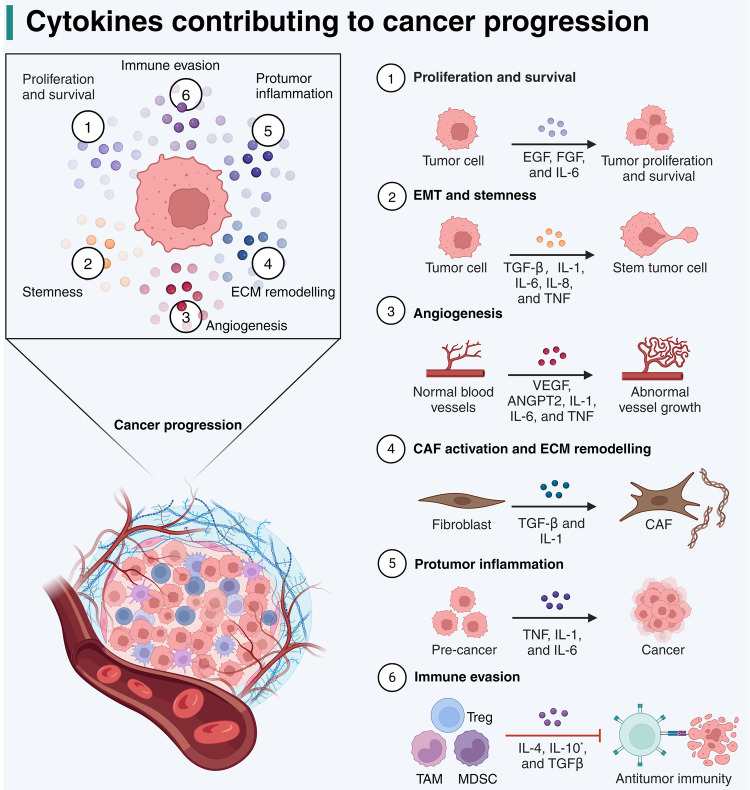
Mechanisms of action of cytokines contributing to cancer progression. This figure illustrates the multifaceted roles of cytokines in cancer. The central diagram shows a tumor microenvironment with key processes labeled 1 through 6, indicating different aspects of cancer progression influenced by cytokines. Firstly, cytokines such as EGF, FGF, and IL-6 promote the proliferation and survival of tumor cells. Secondly, TGF-β, IL-1, IL-6, IL-8, and TNF contribute to the epithelial-mesenchymal transition (EMT) and maintenance of stemness in tumor cells, facilitating a more invasive phenotype. Thirdly, VEGF, ANGPT2, IL-1, IL-6, and TNF drive the formation of new blood vessels (angiogenesis), supplying the tumor with nutrients and oxygen. Moreover, TGF-β and IL-1 are involved in activating fibroblasts to cancer-associated fibroblasts (CAFs) and in extracellular matrix (ECM) remodeling, which promotes tumor immune evasion and treatment resistance. Fifthly, proinflammatory cytokines like TNF, IL-1, and IL-6 create the dysregulated inflammation that can support tumor development and progression. Lastly, anti-inflammatory cytokines including IL-4, IL-10, and TGF-β are implicated in the suppression of CD8^+^ T cell activity and the accumulation of regulatory T cells (Treg), myeloid-derived suppressor cells (MDSC), and tumor-associated macrophages (TAM), which help the tumor evade immune surveillance. Notably, IL-10 generally suppresses immune response, but some studies suggest that it promotes the activation of tumor-resident CD8^+^ T cells. Adapted from “The Tumor Microenvironment: Overview of Cancer-Associated Changes”, by BioRender.com (2024). Retrieved from https://app.biorender.com/biorender-templates

Cytokines, which are typically polypeptides or glycoproteins with relatively small molecular weights (usually in the range of 6 to 70 kDa), regulate the functions, differentiation, proliferation, apoptosis, and survival of their target cells.^1^ When cytokines bind to receptors on target cells, they trigger intracellular signaling pathways to modulate gene transcription, thereby modifying various biological activities. Target cells expressing specific sets of receptors interpret the information from different cytokines based on their concentration and timing of exposure.^2^ Diverse classes of cytokines, including interferons (IFNs), interleukins (ILs), tumor necrosis factor (TNF) superfamily, chemokines, and growth factors, play pivotal roles in homeostasis and diseases.^3^ It is well-established that an imbalanced cytokine profile contributes to cancer initiation and progression by inciting chronic inflammation and immune evasion (Fig. 1).^4^ Consequently, the manipulation or neutralization of abnormal cytokines in the tumor microenvironment (TME) presents a promising approach for the treatment of cancer patients.^5,6^

**Fig. 2:**
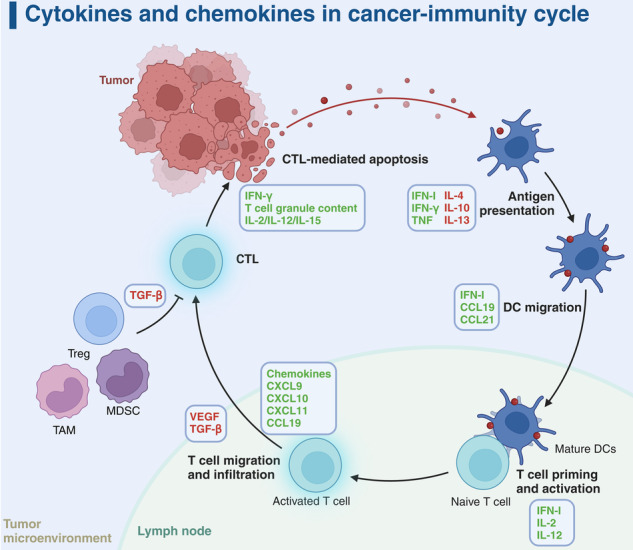
Cytokine dynamics in the cancer-immunity cycle. The figure presents a comprehensive view of cytokine interactions within the cancer-immunity cycle, illustrating the dual role of cytokines in both tumor suppression and promotion. Key features include the promotion of cytotoxic T lymphocyte (CTL)-mediated apoptosis by IFN-γ and various interleukins (IL-2, IL-12, IL-15) within the tumor microenvironment. In contrast, regulatory elements such as regulatory T cells (Tregs), myeloid-derived suppressor cells (MDSCs), and tumor-associated macrophages (TAMs) secrete IL-10 and transforming growth factor-beta (TGF-β) to mitigate CTL efficacy and assist in immune evasion. The lymph node emerges as a pivotal site for antigen presentation by dendritic cells (DCs), orchestrated by a suite of cytokines including type I interferon (IFN-I), IFN-γ, tumor necrosis factor (TNF), along with IL-4, IL-10, and IL-13. DC migration to lymph nodes, necessary for T cell priming and activation, is enhanced by IFN-I, chemokine (C-C motif) ligand 19 (CCL19), and CCL21. Subsequently, activated T cells are drawn back to the tumor via a gradient of chemokines, including C-X-C motif chemokine ligand 9 (CXCL9), CXCL10, CXCL11, and CCL19. Nonetheless, the tumor microenvironment, influenced by vascular endothelial growth factor (VEGF) and TGF-β, can counteract T cell infiltration and activation, underscoring the delicate equilibrium between immune defense and tumor immune evasion. Cytokines are distinctly labeled with red and green to denote their immunosuppressive and immunostimulatory functions for antitumor immunity, respectively. Adapted from “Tumor-Specific T Cell Induction and Function”, by BioRender.com (2024). Retrieved from https://app.biorender.com/biorender-templates

Several cytokines, including IFN-α, IFN-γ, IL-2, IL-12, IL-15, and granulocyte-macrophage colony-stimulating factor (GM-CSF), exhibit antitumor properties in preclinical models.^7^ These cytokines slow tumor growth either by directly inhibiting proliferation and promoting apoptosis, or indirectly by mobilizing an antitumor immune response. For example, IFN-α, originally recognized for its capacity to interfere with viral replication, was discovered to possess antitumor potential five decades ago.^8^ It is now widely accepted that IFN-α not only exerts cytostatic, cytotoxic, and anti-angiogenic effects on tumors but also enhances tumor antigen presentation, primes and activates T cells, boosts the cytotoxic activity of natural killer (NK) cells, improves the maturation and functions of dendritic cells (DCs), and reduces the accumulation of regulatory T cells (Tregs) (Fig. 2).^9^ The positive outcomes in preclinical studies have fostered exploration into employing these cytokine-based immunotherapies for patients with solid and hematologic malignancies. Currently, the Food and Drug Administration (FDA) has granted approval for IFN-α and IL-2 in the treatment of a wide spectrum of cancers, including melanoma, follicular lymphoma, hairy cell leukemia, acquired immunodeficiency syndrome (AIDS)-associated Kaposi’s sarcoma, and renal cell carcinoma.^10–15^ Nevertheless, in clinical practice, these cytokines have largely been superseded by alternative immunotherapies, particularly immune checkpoint blockade (ICB), which offers superior efficacy and more favorable safety profiles.^16–18^ Nonetheless, the potential of combining cytokines with other immunotherapies, along with advances in drug delivery and protein engineering, has reignited interest in cytokines as agents against cancer.^19^

On the contrary, certain cytokines could be hijacked to facilitate cancer progression, such as epidermal growth factor (EGF), vascular endothelial growth factor (VEGF), transforming growth factor-beta (TGF-β), TNF-α, IL-1β, IL-6, colony stimulating factor-1 (CSF-1), C-C motif chemokine ligand 2 (CCL2), CCL5, and C-X-C motif chemokine ligand 8 (CXCL8).^20^ These protumor cytokines actively contribute to various aspects of cancer development, such as growth, metastasis, extracellular matrix remodeling, immune evasion, and resistance to treatment.^21^ Consequently, the neutralization of these protumor cytokines or the blockade of their receptors could potentially enhance the effectiveness of cancer immunotherapy. Currently, several strategies for blocking these cytokines have been developed, encompassing neutralizing antibodies, bispecific antibodies, small-molecule inhibitors, cytokine traps, small interfering RNA (siRNA), and polypeptides.^3^ Some cytokine antagonists, like anti-TGF-β and anti-VEGF antibodies, have shown significant promise in augmenting various immunotherapies, particularly ICB, and alleviating treatment resistance.^22,23^ It is essential to note that most cytokines exhibit versatility, playing diverse roles during different stages of tumor development. As a result, precise patient selection is a crucial prerequisite for optimizing cytokine-targeted therapies. In this comprehensive review, we provide an overview of the role of cytokines in cancer progression, with a particular focus on their involvement in immune evasion. Additionally, we highlight combination strategies involving cytokines or their antagonists, drawing from both preclinical and clinical studies.

## Interferons and their agonists

### Type 1 IFN (IFN-I)

#### The biology of IFN-I

IFN-Is stand as a pivotal group of proteins central to the immune response to a wide array of challenges.^24^ Among these, subtypes like IFN-α and IFN-β interact with a receptor complex, IFNR, composed of IFNαR1 and IFNαR2. This interaction sets off a cascade of signaling events involving Janus kinase 1 (JAK1) and tyrosine kinase 2 (TYK2), triggering the phosphorylation of signal transducer and activator of transcription (STAT)1 and STAT2. Beyond STAT1 and STAT2, IFN-Is also engage other Stat proteins, mitogen-activated protein kinases (MAPK), and phosphatidylinositide 3-kinase (PI3K), thereby activating various IFN regulatory factors (IRFs) and IFN-stimulated genes (ISGs).^25^ These processes create an inflammatory environment conducive to immune clearance.

In the context of cancer, IFN-Is have traditionally been viewed as beneficial, as they have shown the capacity to induce senescence, halt the cell cycle, and promote apoptosis in tumor cells, while also enhancing the antitumor T cell response.^26^ IFN-Is play a pivotal role in supporting cytotoxic T lymphocytes (CTLs) through various mechanisms.^27^ They enhance DC maturation, facilitate antigen presentation, and promote DC migration to lymph nodes, thereby enhancing cross-priming.^28^ IFN-Is augment the effector functions of immune cells, increase the expression of cytotoxic molecules, and facilitate the survival of memory CTLs.^29–31^ Additionally, they prevent the elimination of activated CTLs by NK cells, reduce the ratio of activating versus inhibitory NK cell receptor ligands expressed by CTLs, and stimulate the release of pro-inflammatory cytokines.^32^ Furthermore, IFN-Is curtail the number and functions of Tregs, partially by disturbing cyclic AMP expression.^33^

Notably, interferon epsilon (IFN-ε), a recently discovered member of the IFN-I family, has been identified as an intrinsic suppressor of ovarian cancer. Discovered later than other members of the IFN-I family, IFN-ε is uniquely characterized by its constitutive expression in the female reproductive tract, where it plays a crucial role in defending against sexually transmitted infections.^34^ Notably, IFN-ε expression decreases as ovarian cancer develops, underscoring its potential protective role against tumor progression.^35^ Detailed investigations into IFN-ε have shed light on its complex antitumor activities, which extend beyond its direct impact on tumor cells, including dose-dependent anti-proliferation and apoptosis induction.^35^ Critically, IFN-ε enhances antitumor immunity, evidenced by the activation of T cells and NK cells and the suppression of myeloid-derived suppressor cells (MDSCs) and Tregs.^35^

However, emerging evidence indicates that the impact of IFN-Is on cancer is complex and significantly influenced by the context. While acute and robust IFN-I responses, typically elicited by chemotherapy, radiation therapy, and targeted therapy, have been documented to suppress malignant cell proliferation, playing a crucial role in tumor immunosurveillance, the scenario drastically changes with persistent, weak, and chronic IFN-I signaling. Such prolonged activation paradoxically promotes tumorigenesis and treatment resistance through various cancer cell-intrinsic and immunological mechanisms.^36^ This dual effect mirrors observations in chronic viral infections where sustained IFN-I signaling not only fails in viral clearance but also shifts from immunostimulation to immunosuppression.

Early and adequate IFN-I production in tumors can stimulate DC activation and T-cell cross-priming within the TME, reinforcing antitumor immune responses. Conversely, suboptimal IFN-Is can inadvertently support cancer progression, notably by upregulating immunosuppressive molecules, including immune checkpoints, thus undermining the effectiveness of antitumor T-cell responses.^37^ Chronic IFN-I signaling further modifies the TME by inducing nitric oxide synthase 2 (NOS2) expression, which fosters the recruitment of MDSCs and Tregs, thereby amplifying local immunosuppression.^38^ Additionally, prolonged IFN-I exposure has been linked to increased IL-6 expression by tumor cells, a pro-inflammatory cytokine often associated with mechanisms that facilitate tumor immune evasion.^39^ Moreover, IFN-Is have been identified as drivers of malignant behaviors, such as epithelial-to-mesenchymal transition (EMT) and stemness in cancer cells, factors known to exacerbate tumor progression and resistance to therapy.^40,41^ This complex interplay underlines the imperative for precise modulation of IFN-I signaling within therapeutic strategies. By leveraging IFN-I’s immunostimulatory potential while circumventing its protumor consequences, it is feasible to overcome treatment resistance and enhance therapeutic outcomes. Notably, many cancer treatment strategies, such as chemotherapy, radiotherapy, targeted therapy, and immunotherapy, highly rely on the activation of IFN-I signaling pathways, especially the cyclic GMP-AMP synthase (cGAS)-stimulator of interferon genes (STING) pathway.^3^

#### IFN-α and engineered IFN-α administration

Given the fundamental importance of IFN-Is in both innate and adaptive immunity, IFN-Is hold remarkable potential in the realm of cancer therapy.^42^ The late 1970s marked the beginning of an extensive wave of clinical research that ultimately led to the approval of IFN-α2a and IFN-α2b, both in their standard and pegylated forms, for the treatment of various cancers.^43^ For example, pegylated IFN-α2b has demonstrated efficacy in melanoma by promoting immune infiltration into tumor beds.^44,45^ Besides, combining pegylated IFN-α with the tyrosine kinase inhibitor imatinib has shown promise in increasing molecular responses among patients with chronic myeloid leukemia (CML).^46,47^ Also, combination therapy involving the administration of IFN-α and ICB has shown synergistic effects in patients with liver cancer and melanoma. This synergy can be attributed to the inhibition of glycolysis in tumor cells and enhanced T-cell activation.^48,49^ These encouraging results have led to over 100 ongoing clinical studies worldwide, assessing the safety and efficacy of recombinant IFN-α in a range of hematological and solid tumors.^50–52^

However, despite the potential of IFN-α, its systemic administration can have paradoxical immunosuppressive effects, accompanied by adverse outcomes such as hepatotoxicity, flu-like symptoms, fatigue, gastrointestinal disorders, and depression.^53^ To mitigate these side effects, innovative strategies aim to deliver IFN-Is specifically to the TME.^54^ One such approach is the development of immunocytokine, where IFNs are linked to monoclonal antibodies to target specific cell populations, including malignant cells or leukocyte subsets.^55^ Also, some novel agents, such as ProIFN, increase the tumor-targeting effect by masking IFN-α with its receptor, linked through a cleavable connector, which can be selectively activated by proteases present in the TME.^56^ Another promising strategy involves the genetic engineering of various cell types to express IFN-Is, enhancing their antitumor activity or supporting immune effector cells.^57,58^ For instance, NK cells genetically engineered to express human IFN-α exhibit improved cytotoxicity against hepatocellular carcinoma cells.^59^ Additionally, direct injection of IFN-α-encoding vectors into tumors has shown promise as well. It has been reported that an adenovirus encoding IFN-α reduces tumor-infiltrating Tregs and promotes the accumulation of Th17 cells in colorectal cancers.^60^

#### Increasing IFN-Is by STING agonist and other agents

**Fig. 3:**
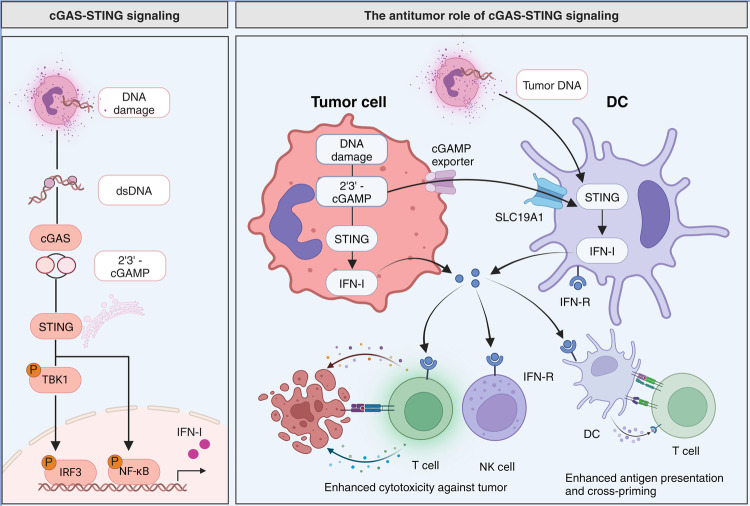
The cGAS-STING signaling pathway and its antitumor effects. The left panel delineates the cGAS-STING signaling cascade initiated by DNA damage, resulting in the production of double-stranded DNA (dsDNA). The enzyme cGAS detects dsDNA and synthesizes 2'3’-cGAMP, which in turn activates STING. Subsequent phosphorylation of TBK1 and IRF3, and activation of NF-κB, leads to the expression of type I interferons (IFN-I). The right panel illustrates the antitumor role of cGAS-STING signaling. Tumor cells undergoing DNA damage could produce 2'3’-cGAMP which activates STING and results in IFN-I release. Besides, tumor-derived DNA and cGAMP can be taken up by dendritic cells (DCs) via the SLC19A1 transporter, leading to STING activation and IFN-I production. Increased IFN-I enhances the cytotoxic activity of T cells and natural killer (NK) cells against the tumor and improves antigen presentation and cross-priming, further promoting T cell activation. (Created with BioRender.com)

The development of tumor-specific adaptive immune responses, including the activation of CD8^+^ T cells with tumor-killing capabilities, relies on IFN-I signaling in antigen-presenting cells (APCs). In the TME, the cGAS-STING signaling pathway represents an evolutionarily conserved innate immune mechanism responsible for regulating the transcription of IFN-I.^61,62^ STING is a cellular DNA sensor located in the endoplasmic reticulum (ER) and is primarily activated by cyclic dinucleotides (CDNs) generated by cGAS rather than direct activation by double-stranded DNA (dsDNA).^63^ Cytosolic dsDNA binds to cGAS, leading to the production of cyclic GMP-AMP (cGAMP) and a change in the conformation of STING (Fig. 3).^64,65^ STING dimers are then translocated from the ER to perinuclear microsomes via the Golgi apparatus. STING recruits and activates TBK1, which phosphorylates IRF3 and upregulates the expression of IFN-I.^66^ STING can also activate the NF-κB pathway by binding to IKK and NIK, collaborating with the TBK1-IRF3 pathway to induce IFN-I expression, which plays a vital role in immune cell maturation and activation.^67^ Pharmacological activation of the cGAS/STING pathway has shown promising results in significantly retarding tumor growth and prolonging the survival of tumor-bearing mice.^68–71^

Besides, accumulated evidence has demonstrated that STING agonists could improve ICB efficacy and overcome immunotherapy resistance.^72–77^ In a phase I clinical trial (NCT03172936), the combination of intratumoral injection of STING agonist ADU-S100 and anti-PD-1 therapy was well tolerated in patients with advanced tumors, with an overall response rate of 10.4%.^78,79^ Besides, intratumoral administration of SYNB1891, a probiotic strain of E. coli engineered to activate STING in the TME, combined with anti-PD-L1 antibody atezolizumab also showed local and systemic safety in patients with advanced or metastatic cancers (NCT04167137).^80^ Moreover, ICB plus intranasal or inhalation administration of natural STING agonist manganese achieved promising efficacy, with the best disease control rate (DCR) of 90.9% and the best objective response rate (ORR) of 45.5%.^81^ Other IFN-I signaling-associated agents, such as polyinosinic-polycytidylic acid (poly I:C) and CpG oligodeoxynucleotide (ODN) multimers, also exhibited the potential to stimulate innate immunity and improve immunotherapy performance (Table 1).^82–86^

**Table 1:** STING agonists for cancer therapy

Category	Agents	Combination partners	Clinical trials	Cancer types	Phase	Status
CDN analog	ADU-S100	Pembrolizumab	NCT03937141	HNSCC	II	Terminated
		Ipilimumab	NCT02675439	Solid tumors or lymphomas	I	Terminated
		PDR001	NCT03172936	Solid tumors or lymphomas	I	Terminated
	MK-1454	Pembrolizumab	NCT04220866	HNSCC	II	Completed
		Pembrolizumab	NCT03010176	Solid tumors or lymphomas	I	Completed
	SB11285	Atezolizumab	NCT04096638	Solid tumors	I	Recruiting
	BMS-986301	Nivolumab or Ipilimumab	NCT03956680	Solid tumors	I	Active, not recruiting
	BI 1387446	Ezabenlimab	NCT04147234	Solid tumors	I	Active, not recruiting
	TAK-676	Pembrolizumab	NCT04879849	Solid tumors	I	Active, not recruiting
		Pembrolizumab	NCT04420884	Solid tumors	I	Recruiting
		Chemotherapy	NCT06062602	HNSCC	I	Completed
Non-CDN	MK-2118	Pembrolizumab	NCT03249792	Solid tumors or lymphomas	I	Completed
	GSK3745417	Monotherapy	NCT05424380	Myeloid malignancies	I	Active, not recruiting
		Dostarlimab	NCT03843359	Solid tumors	I	Active, not recruiting
	Manganese	Radiotherapy	NCT04873440	Solid tumors or lymphomas	I/II	Unknown
		Anti-PD-1	NCT03991559	Solid tumors or lymphomas	I	Unknown
	E7766	Monotherapy	NCT04144140	Solid tumors or lymphomas	I	Terminated
		Monotherapy	NCT04109092	Bladder cancer	I	Withdrawn
	SNX281	Pembrolizumab	NCT04609579	Solid tumors or lymphomas	I	Terminated
Engineered bacteria	SYNB1891	Atezolizumab	NCT04167137	Solid tumors or lymphomas	I	Unknown
ADC	TAK-500	Pembrolizumab	NCT05070247	Solid tumors	I	Recruiting
	XMT-2056	Monotherapy	NCT05514717	Her-2 positive solid tumors	I	Recruiting

Note: *ADC* antibody-drug conjugate, *CDN* cyclic dinucleotide, *HNSCC* head and neck squamous cell carcinoma. The specifics of the clinical trials were sourced in January 2024 from the ClinicalTrials.gov website

### IFN-γ

#### IFN-γ signaling and its dual role in cancer

IFN-γ, the exclusive member of the IFN-II family, plays a versatile role encompassing antiviral, antitumor, and immunomodulatory functions. It holds a central position in orchestrating both innate and adaptive immune responses.^87^ Within an inflammatory milieu, IFN-γ contributes to activating the immune response, aiding pathogen clearance, while also preventing excessive immune activation and tissue damage.^88^ In the TME, IFN-γ exhibits both protumor and antitumor activities, which are largely dependent on the duration and magnitude of the signaling.^89^ Initially identified as a cytotoxic cytokine, along with perforin, granzyme, and TNF, IFN-γ is known for inducing apoptosis in tumor cells.^90,91^ Furthermore, IFN-γ can impede angiogenesis in tumors, induce apoptosis in Tregs, improve the maturation of DCs, and enhance the activity of M1-like macrophages, effectively impeding tumor progression.^92^ Generally, given its cytostatic, pro-apoptotic, and anti-proliferative properties, IFN-γ emerges as a promising candidate for adjuvant immunotherapy in diverse cancers (Table 2). However, recent studies have revealed the antitumor effect of IFN-γ. Similar to IFN-Is, prolonged IFN-γ exposure facilitates the upregulation of immune inhibitory molecules such as PD-L1, PD-L2, CTLA-4, and indoleamine-2,3-dioxygenase (IDO), thus promoting cancer immune evasion.^93^ Additionally, some tumor cells evade the antitumor effects of IFN-γ through modifications in the receptor or downstream JAK/STAT signaling pathway, alongside the constitutive activation of JAK inhibitors such as SOCS1 and SOCS3.^94^

**Table 2:** Clinical trials harnessing IFN-γ for cancer therapy

NCT number	Cancer types	Combination partners	Phase	Status
NCT03112590	HER2-positive Breast Cancer	Paclitaxel, Trastuzumab, and Pertuzumab	I/II	Completed
NCT00002637	Prostate Cancer	Gene-modified tumor cell vaccine therapy	I/II	Completed
NCT00786643	Colorectal Cancer	5-Fluorouracil, Leucovorin, and Bevacizumab	II	Completed
NCT00002796	Colorectal Cancer	Fluorouracil, Sodium phenylbutyrate, and Indomethacin	I/II	Terminated
NCT00047632	Ovarian/Peritoneal Carcinoma	Monotherapy	III	Terminated
NCT00001296	Melanoma	Melphalan and TNF	III	Completed
NCT00501644	Ovarian/Fallopian Tube/Peritoneal Cancer	Carboplatin and GM-CSF	II	Completed
NCT00002505	Solid Tumors	Tumor cell lysate vaccine	II	Completed
NCT00616720	Multiple Myeloma and Plasma Cell Neoplasm	Autologous dendritic cell vaccine APC8020	II	Completed
NCT01082887	Melanoma	Adoptive transfer of TIL and IFN-γ-adenovirus	I/II	Terminated
NCT00057447	Non-Hodgkin’s Lymphoma	Rituximab	I/II	Terminated
NCT00394693	B-Cell Lymphoma	IFN-γ-adenovirus	II	Completed
NCT00002475	Solid Tumors	Allogeneic tumor cell vaccine and cyclophosphamide	II	Completed
NCT00070187	Lymphoma	Aldesleukin, Filgrastim, Chemotherapy, and Bone marrow transplantation	II/III	Completed
NCT02380443	Colorectal Cancer	In-Situ Cancer Vaccine, and Cryoablation	II	Completed
NCT00006113	Melanoma	Cancer vaccine therapy, and Aldesleukin	II	Terminated
NCT00024271	Malignant Mesothelioma	Surgery, Chemotherapy, and Radiation therapy	II	Unknown
NCT02550678	Skin Neoplasm	ASN-002 (adenovirus) and 5-FU	I/II	Completed
NCT00002761	Leukemia	Aldesleukin, Filgrastim, Chemotherapy, and Bone marrow transplantation	I/II	Withdrawn

Note: *TIL* tumor-infiltrating lymphocyte, *GM-CSF* granulocyte-macrophage colony-stimulating factor, *TNF* tumor necrosis factor

#### IFN-γ therapy

In both basic and clinical investigations, IFN-γ has emerged as a factor contributing to the direct or indirect eradication of tumors through collaboration with other components of the TME. The intraperitoneal administration of recombinant human IFN-γ yielded a 23% complete regression (CR) rate in ovarian cancer patients with residual diseases.^95^ In the first-line therapy for ovarian cancer, the combination of chemotherapy with subcutaneous IFN-γ treatment demonstrated a superior therapeutic efficacy compared to chemotherapy alone. Key outcomes included a 3-year progression-free survival (PFS) rate of 51% versus 38%, median times to progression of 48 versus 17 months, and a complete clinical response rate of 68% versus 56%.^96^ However, in expansive phase III clinical trials involving advanced ovarian and primary peritoneal carcinomas, IFN-γ failed to confer additional survival benefits. Instead, interim analysis revealed that patients receiving chemotherapy combined with subcutaneous IFN-γ therapy experienced a shorter overall survival (OS) and an elevated risk of serious hematological toxicities.^97^ Furthermore, the administration of IFN-γ in various other cancers, including renal-cell carcinoma, melanoma, and colon cancer, did not achieve positive results.^98–100^ Given its generally modest clinical efficacy, IFN-γ treatment has not gained approval for any solid tumor indication. These findings underscore the nuanced and context-dependent nature of therapeutic effects of IFN-γ, emphasizing the need for a cautious approach in its application for solid tumor indications.

Significantly, IFN-γ is recognized as a pivotal determinant for the success of immunotherapy. Recent advances highlight the critical role of interferon-γ receptor (IFNγR) signaling in modulating the efficacy of chimeric antigen receptor (CAR) T cell therapy, particularly in solid tumors. A pivotal study employing a genome-wide CRISPR knockout screen revealed a marked increase in resistance to CAR-T cell therapy in solid tumors upon disruption of key genes within the IFNγR signaling pathway, such as *IFNGR1*, *JAK1*, or *JAK2*.^101^ This phenomenon is notably absent in hematologic malignancies like leukemia and lymphoma, underscoring a distinct mechanism of interaction between CAR-T cells and solid tumor cells.^101^ Specifically, the study illuminated that *IFNGR1*-deficient glioblastoma cells exhibited significantly reduced adhesion and subsequent cytotoxicity by CAR-T cells.^101^ This finding stresses the indispensability of IFNγR signaling for the effective targeting of solid tumors by CAR-T therapy. Also, in patients responsive to anti-PD-1 therapy, there was a notable upregulation of the IFN-γ-related gene signature, distinguishing them from non-responders.^102–104^ Moreover, resistance to anti-CTLA-4 in melanoma patients is often associated with deficiencies in the IFN-γ pathway, including the loss of *IFNGR*, *JAK2*, *IFIT*, *MTAP*, and *IRF1* genes. In murine melanoma models, silencing the *IFNGR1* gene nullified the efficacy of anti-CTLA-4.^105^ IFN-γ has been validated as a promoter of T cell infiltration, upregulating major histocompatibility complex class (MHC) and PD-L1 expression in tumors while limiting the accumulation of immunosuppressive components, such as CXCR2^+^CD68^+^ macrophages, in the TME.^106,107^ Consequently, it is rational to combine IFN-γ with anti-PD-1/PD-L1 for optimal cancer immunotherapy. In a phase I study (NCT02614456), the combination of IFN-γ and nivolumab exhibited modest clinical benefits, with an ORR of 4.3% and a DCR of 26.1% in advanced solid tumors.^108^ Presently, several ongoing clinical studies are exploring the effects of systemic IFN-γ therapies.^109^

## Interleukins and their agonists or inhibitors

### IL-2

#### IL-2 signaling and its role in cancer immunology

**Fig. 4:**
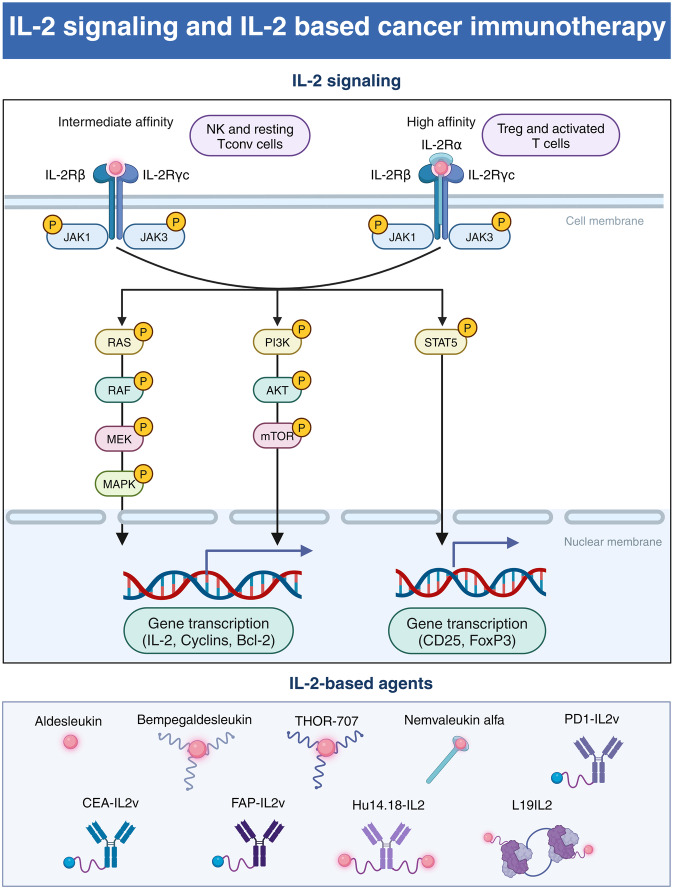
IL-2 signaling pathway and IL-2 based cancer immunotherapy. This schematic representation illustrates the differential signaling pathways activated by the binding of IL-2 to its receptor complexes of varying affinities. On the left, the intermediate affinity IL-2 receptor, composed of IL-2Rβ and IL-2Rγc subunits, is primarily found on natural killer (NK) cells and resting conventional T (Tconv) cells. On the right, the high affinity IL-2 receptor, which includes the IL-2Rα subunit in addition to IL-2Rβ and IL-2Rγc, is expressed on regulatory T (Treg) cells and activated T cells. The binding of IL-2 to it receptor activates the RAS/RAF/MEK/MAPK, PI3K/AKT/mTOR and JAK/STAT pathways, which leads to gene transcription of IL-2, Cyclins, and Bcl-2, CD25 and FoxP3. The lower portion of the figure showcases a selection of IL-2-based agents used in cancer immunotherapy, including Aldesleukin, Bempegaldesleukin, THOR-707, Nemvaleukin alfa, PD1-IL2v, CEA-IL2v, FAP-IL2v, Hu14.18-IL2, and L19IL2, each designed to leverage the IL-2 signaling pathways for therapeutic purposes. (Created with BioRender.com)

IL-2, initially identified in the supernatants of activated T cells and formerly labeled as T-cell growth factor, plays a pivotal role in immune regulation.^110^ The IL-2 receptor is a trimeric complex consisting of IL-2Rα (CD25), IL-2Rβ (CD122), and IL-2Rγ (CD132), each exhibiting distinct affinities for IL-2. IL-2 demonstrates low affinity for IL-2Rα, intermediate affinity for IL-2Rβ and IL-2Rγ, and high affinity for heterotrimeric receptors containing all three subunits.^111^ Generally, Tregs primarily express the high-affinity trimeric IL-2 receptor, whereas CD8^+^ T cells and NK cells predominantly express the intermediate-affinity dimeric IL-2 receptor (IL-2Rβ/γ complex).^112^ The interaction between IL-2 and IL-2R triggers downstream JAK-STAT, MAPK, and PI3K signaling pathways by the intracellular domains of IL-2Rβ/γ complex (Fig. 4).^113^ It has been well established that IL-2 is a core cytokine maintaining adaptive immunity. Primarily, IL-2 promotes the proliferation, differentiation, and cytotoxic activity of T cells.^114,115^ Also, IL-2 contributes to immune homeostasis by supporting the expansion of Tregs.^116^ Accumulating evidence underscores the critical role of IL-2 in cancer immunology. Impaired IL-2 signaling is associated with poor outcomes in various cancers, while IL-2-based therapies show promise in stimulating antitumor immune response and improving immunotherapy efficacy in cancer patients.^117,118^

#### IL-2 and engineered IL-2 administration

In 1985, Steven Rosenberg first reported a patient with metastatic melanoma experiencing complete regression despite severe toxicities after high-dose intravenous IL-2 treatment.^119^ Subsequent studies confirmed the antitumor potential of high-dose IL-2 in patients with metastatic melanoma and renal cell cancers.^120^ These positive data led to FDA approval of high-dose IL-2 therapy for metastatic renal cell carcinoma in 1992 and metastatic melanoma in 1998.^121^ Despite its efficacy, high-dose IL-2 therapy has limitations, including a short half-life and severe toxicities such as vascular leak syndrome. Besides, patients not responding to high-dose IL-2 exhibited increased Treg cell expansion, which might promote tumor progression in turn.^122^ To address these limitations, new therapies are being designed to selectively enhance immune activation but avoid Treg accumulation and high IL-2 dosing.

The next generation of IL-2-based antitumor agents has biased affinities to IL-2R subunits **(**Table 3**)**. For instance, Bempegaldesleukin, a PEGylated IL-2 variant, selectively activates CD8^+^ T cells and NK cells by preferentially binding to IL-2Rβγ, minimizing impact on Tregs. This PEGylation modification aims to reduce toxicities and extend IL-2 half-life.^123^ In the phase I study, Bempegaldesleukin induced tumor regression in advanced or metastatic solid tumors as a monotherapy.^124^ Combined with nivolumab, it achieves an ORR of 59.5% and a complete response rate of 18.9% in immunotherapy-naïve advanced solid tumors, with tolerable adverse events.^125^ Besides, Nemvaleukin alfa, an engineered fusion protein comprising IL-2 and the extracellular domain of IL-2Rα, is designed to selectively activate effector lymphocytes by binding to intermediate-affinity IL-2 receptors, excluding high-affinity receptors present on Tregs and endothelial cells.^126^ The protein’s preferential expansion of CD8^+^ T cells and NK cells, coupled with minimal expansion of immunosuppressive Tregs, underscores its potential to evoke robust systemic antitumor immunity^127^. Preclinical models demonstrated the outstanding antitumor efficacy of Nemvaleukin alfa, whether administered intravenously or subcutaneously, showcasing superior effectiveness and a notable reduction in distant metastasis.^128,129^ In a phase I/II clinical trial (NCT02799095), both Nemvaleukin alfa monotherapy and its combination with pembrolizumab exhibited promising antitumor activity in patients with advanced solid tumors.^130^

**Table 3:** Clinical trials harnessing IL-2 and its engineered variants for cancer therapy

Products	NCT number	Cancer types	Combination partners	Phase	Status
Aldesleukin	NCT00018941	Kidney Cancer	Monotherapy	III	Completed
	NCT00416871	Kidney Cancer	IFN-α	III	Completed
	NCT00002702	Head and Neck Cancer	Surgery and Radiation Therapy	III	Unknown
	NCT00003126	Kidney Cancer	Monotherapy	III	Completed
	NCT00039234	Melanoma	Histamine Dihydrochloride	III	Unknown
Nemvaleukin alfa	NCT04592653	Solid Tumors	Pembrolizumab	I/II	Recruiting
	NCT05092360	Ovarian/Fallopian Tube/Peritoneal Cancer	Pembrolizumab	III	Recruiting
	NCT03861793	Solid Tumors	Pembrolizumab	I/II	Completed
	NCT02799095	Solid Tumors	Pembrolizumab	I/II	Completed
	NCT04144517	HNSCC	Pembrolizumab	II	Completed
	NCT04830124	Melanoma	Monotherapy	II	Recruiting
Bempegaldesleukin	NCT03785925	Bladder Cancer	Nivolumab	II	Completed
	NCT03548467	Solid Tumors	VB10.NEO	I/II	Completed
	NCT04209114	Bladder Cancer	Nivolumab	III	Completed
	NCT04969861	HNSCC	Pembrolizumab	II/III	Terminated
	NCT04052204	HNSCC and mCRPC	Avelumab, Talazoparib, and Enzalutamide	I/II	Terminated
	NCT03138889	NSCLC	Pembrolizumab and Chemotherapy	I/II	Terminated
	NCT04730349	Solid tumors	Nivolumab	I/II	Terminated
	NCT03435640	Solid tumors	NKTR-262 and Nivolumab	I/II	Terminated
	NCT04936841	HNSCC	Radiation and Pembrolizumab	II	Terminated
	NCT03745807	Solid tumors	Nivolumab	I	Completed
	NCT02983045	Solid tumors	Nivolumab	I/II	Completed
	NCT04540705	RCC	Nivolumab	I	Active, not recruiting
	NCT03729245	RCC	Nivolumab	III	Terminated
	NCT04410445	Melanoma	Nivolumab	III	Terminated
	NCT03635983	Melanoma	Nivolumab	III	Completed
THOR-707	NCT04914897	Pleural Mesothelioma and NSCLC	Pembrolizumab	II	Active, not recruiting
	NCT04009681	Solid tumors	ICB and Anti-EGFR antibody	I/II	Recruiting
	NCT05104567	Gastrointestinal Cancers	Pembrolizumab and Cetuximab	II	Active, not recruiting
	NCT04913220	Skin Cancers	Cemiplimab	I/II	Active, not recruiting
	NCT05061420	HNSCC	Pembrolizumab and Cetuximab	II	Active, not recruiting
	NCT05179603	Lymphoma	Pembrolizumab	II	Active, not recruiting
RO6895882 (CEA-IL2v)	NCT02004106	Solid Tumors	Monotherapy	I	Completed
	NCT02350673	Solid Tumors	Atezolizumab	I	Completed
Eciskafusp alfa (PD1-IL2v)	NCT04303858	Solid Tumors	Atezolizumab	I	Recruiting
Simlukafusp alfa (FAP-IL2v)	NCT03386721	Solid Tumors	Atezolizumab, Gemcitabine, and Vinorelbine	II	Terminated
	NCT02627274	Solid Tumors	Trastuzumab and Cetuximab	I	Completed
	NCT03875079	Melanoma	Pembrolizumab	I	Completed
	NCT03063762	RCC	Atezolizumab and Bevacizumab	I	Completed
L19IL2	NCT01198522	Pancreatic Cancer	Gemcitabine	I	Terminated
	NCT01058538	Solid Tumors	Monotherapy	I/II	Completed
	NCT02086721	Solid Tumors	Monotherapy	I	Completed
	NCT05329792	Skin Cancers	L19TNF	II	Recruiting
	NCT02735850	NSCLC	Radiotherapy	II	Withdrawn
	NCT04362722	Skin Cancers	L19TNF	II	Recruiting
	NCT02076646	Melanoma	Dacarbazine	I/II	Active, not recruiting
	NCT02957019	DLBCL	Rituximab	I/II	Terminated
	NCT01055522	Melanoma	Dacarbazine	II	Terminated
	NCT03705403	NSCLC	Radiation	II	Unknown
	NCT01253096	Melanoma	Monotherapy	II	Completed
	NCT02076633	Melanoma	L19TNF	II	Completed
	NCT02938299	Melanoma	L19TNF	III	Recruiting
	NCT03567889	Melanoma	Monotherapy	III	Recruiting
Hu14.18-IL2	NCT00003750	Melanoma	Monotherapy	I	Completed
	NCT00590824	Melanoma	Monotherapy	II	Completed
	NCT00109863	Melanoma	Monotherapy	II	Completed
	NCT03209869	Neuroblastoma	Donor NK Cell	I	Withdrawn
	NCT00082758	Neuroblastoma	Monotherapy	II	Completed
	NCT01334515	Neuroblastoma	Sargramostim and Isotretinoin	II	Completed

Note: *HNSCC* head and neck squamous cell carcinoma, *NSCLC* non-small cell lung cancer, *RCC* renal cell cancer, *mCRPC* metastatic castration resistant prostate cancer, *DLBCL* diffuse large B-cell lymphoma

Moreover, some recent studies reported the potent antitumor effects of an engineered variant of IL-2 (IL-2v), specifically PD1-IL2v, in various preclinical tumor models.^131–133^ PD1-IL2v demonstrates multifaceted molecular mechanisms of action, including targeting IL-2v to PD-1^+^ tumor-specific T cells, IL-2Rα-independent binding to IL-2R, prolonged interaction with IL-2R through PD-1 anchoring, and partial PD-1 signaling blockade.^134^ Single-cell RNA-seq data have demonstrated that PD1-IL2v treatment increases the frequency of optimally activated T cells, particularly tumor-infiltrating GZMB^+^TIM-3^−^PD-1^+^TCF7^−^CD8^+^ cells.^135^ Additionally, TransCon IL-2β/γ, a sustained-release drug of IL-2Rβ/γ-selective IL-2v, effectively increased the proliferation and cytotoxicity of primary CD8^+^ T cells, NK cells, and γδ T cells without severe toxicities, especially vascular leak syndrome and cytokine storm.^136^ Generally, the selective expansion of CD8^+^ T cells and NK cells, alongside a manageable safety profile, positions IL-2-based therapy as a compelling therapeutic candidate in the dynamic realm of immunotherapy for advanced solid tumors.

### IL-10

#### The dual role of IL-10: general immunosuppression but tumor-resident CD8^+^ T cell activation

IL-10, a dimeric protein encoded by the *IL10* gene on chromosome 1, is primarily produced by a variety of immune cell types, including T cells, B cells, NK cells, and mast cells.^137^ Notably, certain tumor cells, such as those associated with human papilloma virus (HPV)-related cervical cancers, can also generate IL-10.^138^ The IL-10 receptor (IL-10R), expressed on hematopoietic cells, comprises two subunits, IL-10Rα and IL-10Rβ, initiating downstream STAT1 or STAT3 signaling through the phosphorylation of JAK1 and Tyk2.^139^ Subsequently, STAT3 translocates to the nucleus, prompting the expression of genes responsive to STAT3, including SOCS3 and IL1RN.^140^ SOCS3 exerts its inhibitory effect on inflammatory gene expression by impeding MAPK and NF-κB pathways, while IL1RN functions as a decoy protein, interfering with IL-1β signaling by binding to its receptor and suppressing inflammatory responses.^141^

In a broader context, IL-10 assumes a pivotal role in curbing excessive inflammatory responses, contributing to immune tolerance, and mitigating autoimmune diseases.^142^ By downregulating MHC-II, IL-10 attenuates DC responses to antigen stimulation, leading to the reduction of various immunostimulatory cytokines.^143^ Furthermore, IL-10 impedes the proliferation and function of CD4^+^ T cells, thereby contributing to an immunosuppressive TME.^144^ Conversely, its impact on CD8^+^ T cells is distinctive,^145^ as preclinical studies indicate its role in activating tumor-resident CD8^+^ T cells, retarding tumor growth in murine tumor models.^146^ IL-10 induces STAT1/3 phosphorylation specifically in tumor-resident CD8^+^ T cells, enhancing IFN-γ expression and granzyme production, thereby promoting an augmented immune response and facilitating antiproliferative and proapoptotic pathways.^146^ These findings have stimulated interest in investigating the therapeutic potential of IL-10 in cancer patients, with emerging results demonstrating promising efficacy in specific tumor types, such as renal cell carcinoma, though its activity in other tumors varies.^147^

#### Engineered IL-10 treatment

Pegilodecakin, the first pegylated form of IL-10, exhibited promising activity and a reasonable safety profile in the phase I trial NCT02009449 (Table 4).^148^ The dose-escalation and -expansion cohorts included 51 patients with various solid tumors, and the drug, administered through daily subcutaneous injections, demonstrated good tolerability with no maximum-tolerated dose reached in the dose-escalation cohort.^148^ Notable adverse events were generally mild, including anemia, fatigue, fever, injection-site reactions, and thrombocytopenia. One patient with uveal melanoma and four out of 15 evaluable patients with RCC exhibited partial responses when treated at a dosage of 20 μg/kg, even in those who had received prior immunotherapy.^148^ In the other two cohorts of phase I trial NCT02009449, Pegilodecakin was combined with anti-PD-1 antibodies (pembrolizumab or nivolumab).^149^ Response rates varied by tumor type, with notable responses observed in NSCLC (ORR: 43%), renal cell carcinoma (ORR: 40%), and melanoma (ORR: 10%).^149^ The combination therapy achieved a favorable response in NSCLC and renal cell carcinoma, but with manageable toxicity of thrombocytopenia and anemia relative to anti-PD-1 monotherapy.^149^ However, in phase II trials (NCT03382899 and NCT03382912), combining Pegilodecakin with anti-PD-1 therapy in metastatic NSCLC did not improve ORR, PFS, or OS compared to anti-PD-1 therapy alone.^150^ The combination led to more frequent overall and serious adverse events.^150^ Similarly, in a phase III trial for pancreatic ductal adenocarcinoma (NCT02923921), the addition of Pegilodecakin to FOLFOX chemotherapy did not improve ORR and survival, while increased adverse events were noted in the combination arm.^151^

**Table 4:** Clinical trials targeting IL-10 for cancer therapy

Products	NCT number	Cancer types	Combination partners	Phases	Status
Pegilodecakin (PEGylated IL-10)	NCT02923921	Pancreatic Cancer	FOLFOX	III	Completed
	NCT03382912	NSCLC	Nivolumab	II	Terminated
	NCT03382899	NSCLC	Pembrolizumab	II	Terminated
	NCT02009449	Solid tumors	Chemotherapy	I	Active, not recruiting
IBB0979 (B7H3-IL10 immunocytokine)	NCT05991583	Solid tumors	Monotherapy	I/II	Recruiting
IAE0972 (EGFR/IL10 immunocytokine)	NCT05396339	Solid tumors	Monotherapy	I/II	Recruiting

Note: *NSCLC* non-small cell lung cancer, *FOLFOX* 5-fluorouracil and oxaliplatin, *EGFR* epidermal growth factor receptor

Several strategies have been explored to enhance the therapeutic potential of IL-10 beyond PEGylation. One approach involved the development of a bispecific fusion protein by combining cetuximab with the IL-10 dimer to enhance drug delivery to tumors expressing epidermal growth factor receptor (EGFR).^152^ This fusion protein exhibited an extended half-life without increased toxicity and demonstrated significant antitumor effects in murine tumor models.^152^ Other IL-10-based strategies, such as engineered IL-10 variants with increased affinity toward IL-10Rβ, incorporating IL-10 into oncolytic viruses, and conjugating IL-10 to nanoparticles, also demonstrated potent antitumor potency.^153–155^ Generally, although IL-10 monotherapy demonstrated good tolerability, its clinical efficacy in large-scale clinical trials was modest. Nevertheless, the exploration of IL-10 in cancer immunotherapy remains a topic of clinical interest, urging further investigation into potential combination strategies or IL-10 modifications.

### IL-12

#### IL-12 signaling and its role in cancer immunology

**Fig. 5:**
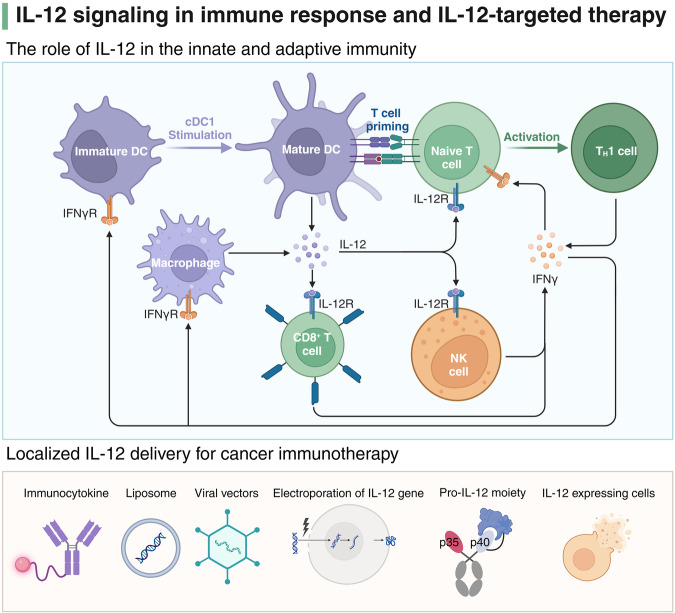
IL-12 signaling pathway and therapeutic applications. The upper panel illustrates the role of IL-12 in both innate and adaptive immunity. When exposed to stimuli such as pathogen-associated molecular patterns, immature dendritic cells (DCs) undergo transformation into their mature form, subsequently leading to the production of interleukin-12 (IL-12). This cytokine mainly acts on T cells and natural killer (NK) cells via the IL-12 receptor (IL-12R). IL-12 is pivotal for T cell priming and the subsequent differentiation of naive T cells into Th1 cells, with IFN-γ acting as a critical feedback enhancer of this immune response. The lower panel depicts strategies for localized IL-12 delivery in cancer immunotherapy, including immunocytokines, liposomes, viral vector, electroporation of the IL-12 gene, pro-IL-12 moieties, and cells engineered to express IL-12. Adapted from “Differentiation of TH17 Cells - Indirect and direct activation of T cells by TLR agonists”, by BioRender.com (2024). Retrieved from https://app.biorender.com/biorender-templates

IL-12 is the first identified member of the IL-12 family, constituted by two distinctive subunits: the p35 α-chain and the p40 β-chain.^156^ Correspondingly, its receptor exhibits a dimeric structure, comprising IL-12Rβ1 and IL-12Rβ2 subunits.^157^ APCs, including DCs, phagocytes, and B cells, primarily produce IL-12. Concurrently, NK and T cells serve as the main targets for IL-12.^158^ APCs, upon detection of pathogen-associated molecular patterns (PAMPs) through toll-like receptors (TLRs), trigger the transcription of IL-12p35 and IL-12p40.^159^ The binding of the IL-12 to the IL-12 receptor subunits initiates the JAK-STAT pathway for signal transduction. Tyrosine kinases JAK2 and TYK2 are recruited and undergo phosphorylation, subsequently phosphorylating the IL-12Rβ2 subunit.^160^ This signaling cascade initiates gene transcription, particularly facilitating STAT4-mediated expression of IFN-γ. It has been substantiated that IL-12 occupies a central role in the differentiation of T helper 1 (Th1) cells and the transcription of IFN-γ in effector cells (Fig. 5).^161^ Conversely, IL-12 hinders the differentiation of Th2 cells by suppressing the Th2-associated transcription factor GATA3 within T cell populations.^162^

#### Innovative IL-12-based therapies: localized IL-12 delivery

Although IL-12 has shown promising antitumor effects in preclinical studies, its efficacy at tolerated doses has been limited in clinical trials (ORR: 5%).^163^ Besides, the later phase II clinical trial of rhIL-12 was halted due to serious safety concerns, with two fatalities reported.^164^ Despite unsatisfactory initial clinical outcomes, IL-12 remained a compelling target for enhancing anti-cancer immunity. Researchers explored various preclinical strategies to improve IL-12-based therapy efficacy while mitigating its systemic toxicity. Numerous approaches have been assessed to achieve localized delivery of IL-12, aiming to maximize IL-12 abundance in the TME and minimize peripheral leakage and toxicity.^165^ At present, some of these novel methods are progressing toward clinical applications.

Various viral vectors, such as adenovirus, adeno-associated virus (AAV), Semliki Forest virus (SFV), and herpes simplex virus (HSV), have been employed for localized IL-12 delivery, demonstrating therapeutic efficacy in murine tumor models.^166–169^ While retroviral vector-based approaches effectively express IL-12 in transfected cells,^170^ their limited use for in vivo applications arises from safety concerns associated with random genome integration.^171^ In response to these concerns, non-integrative vectors such as adenovirus and AAV have been developed, which exhibit promise in preclinical models and are undergoing clinical evaluation.^172,173^ A notable advantage conferred by viral vectors resides in the capacity of oncolytic viruses to kill tumor cells directly. Furthermore, viral infection could activate PAMPs and enhance the functions of APCs, further improving antitumor immune response.^165^ Moreover, an alternative method for inducing localized expression of IL-12 involves the use of non-viral vectors. This includes the administration of nucleic acids, either in their naked form or intricately combined with polymers or lipid-based delivery systems.^174,175^ Multiple phase I/II clinical trials, utilizing in-vivo electroporation for IL-12 gene transfer, documented heightened IFN-γ level, increased infiltration of T cells, and effective tumor control in patients with ovarian cancer and melanoma (Table 5).^176–178^ Furthermore, combination therapy of IL-12 plasmid (Tavo) and pembrolizumab yielded promising outcomes in patients with metastatic melanoma.^179^ Apart from DNA, mRNA-based IL-12 delivery, particularly using lipid nanoparticles (LPNs), has proven both safe and effective in preclinical models.^180^ Notably, human IL-12 mRNA LPN products like MEDI1191 have progressed into clinical trials.

**Table 5:** Clinical trials involving IL-12 for cancer therapy

Products	NCT number	Cancer types	Combination partners	Phases	Status
M032	NCT02062827	Glioblastoma	Monotherapy	I	Active, not recruiting
	NCT05084430	Glioblastoma	Pembrolizumab	I/II	Recruiting
MEDI1191	NCT03946800	Solid Tumors	Durvalumab	I	Completed
MEDI9253	NCT04613492	Solid Tumors	Durvalumab	I	Active, not recruiting
M9241	NCT05361798	Prostate Cancer	SBRT	II	Recruiting
	NCT06096870	Prostate Cancer	Enzalutamide	II	Not yet recruiting
	NCT04633252	Prostate Cancer	Docetaxel	I/II	Recruiting
	NCT04327986	Pancreatic Cancer	M7824 and SBRT	I/II	Terminated
	NCT04235777	Genitourinary Malignancies	M7824 and SBRT	I	Recruiting
	NCT05286814	Colorectal Cancer or Intrahepatic Cholangiocarcinoma	Chemotherapy	II	Recruiting
	NCT02994953	Solid Tumors	Avelumab	I	Terminated
	NCT04756505	Breast Cancer	M7824 and SBRT	I	Withdrawn
	NCT04708470	HPV-Associated Malignancies, Small Bowel, and Colon Cancers	M7824 and Entinostat	I/II	Recruiting
	NCT04491955	Small Bowel and Colorectal Cancers	CV301, MSB0011359C, and N-803	II	Active, not recruiting
SAR441000	NCT03871348	Solid Tumors	Cemiplimab	I	Active, not recruiting

Note: *SBRT* stereotactic body radiation therapy, *HPV* human papillomavirus

Moreover, immunocytokines represent an innovative strategy for targeted IL-12 delivery to the TME. Most immunocytokine products are chimeric constructs combining an antibody with a cytokine, with the cytokines fused either to the N-term or the C-term of complete IgG antibodies or smaller antibody fragments.^181^ Several IL-12 immunocytokines, such as BC1-IL12 and NHS-IL12, are now undergoing clinical trials. BC1-IL12 utilizes the single chain fragment variable (scFv) of the L19 antibody (recognizing fibronectin) to target the TME,^182^ while NHS-IL12, created using the NHS76 antibody (targeting DNA-histone complexes), shows efficacy in inducing tumor regression.^183^ Additionally, pro-cytokines, where IL-12 is shielded by peptides and unmasked by matrix metalloproteinase 9 (MMP9) in the TME, present another avenue.^184^ The pro-IL-12 moiety, employing an MMP14 cleavable substrate linker, has shown localized cleavage and accumulation of active IL-12 in the tumor bed, displaying robust efficacy in controlling murine tumor growth.^185^ Furthermore, engineered mutant forms of IL-12 p40 retain antitumor activity while exhibiting enhanced safety, showcasing diverse and promising strategies in the development of IL-12-based therapies for cancer treatment.^186^

### IL-15

#### IL-15 vs. IL-2: shared receptors with unique trans-presentation mode

IL-15 is a member of the four-α-helix bundle cytokine family, alongside cytokines such as IL-2, IL-4, and IL-7.^187^ A distinguishing feature of IL-15 within this family lies in its trans-presentation mode. Commonly, IL-15 exists stably in conjunction with its high-affinity receptor α (IL-15Rα), forming IL-15/IL-15Rα complex on APCs.^188^ Under this circumstance, IL-15 is trans-presented by IL-15Rα to target cells, including NK, NKT, and memory CD8^+^ T cells, by binding IL-2Rβ/γc receptor complex.^189^ While the predominant IL-15 signaling pathway involves the IL-15/IL-15Rα complex, IL-15 could independently bind to the IL-2Rβ/γc complex in the absence of IL-15Rα, with lower binding affinity.^190^ Upon activation, the β and γc chains initiate intracellular JAK-STAT signaling.^191^ Despite IL-15 and other four-helix bundle cytokines engaging with common receptor subunits, the unique trans-presentation mode contributes to IL-15’s distinctive functions. For example, both IL-15 and IL-2 bind to and stimulate NK and CD8^+^ T cells, but Tregs are primarily stimulated by IL-2.^192^ Furthermore, in contrast to IL-2, IL-15 plays a critical role in inhibiting activation-induced cell death, thereby promoting the survival of memory cells.^193^ It has been confirmed that IL-15 is indispensable for the proliferation, maintenance, and survival of NK and CD8^+^ T cells.^194^

#### IL-15 and engineered IL-15 treatment

The potential of recombinant human IL-15 (rhIL-15) has been extensively explored as an immunomodulator against cancers. In preclinical studies, rhIL-15 has demonstrated superiority over IL-2 in reducing tumor burden and prolonging survival in tumor-bearing mice.^195^ In patients with renal cell carcinoma and melanoma, rhIL-15 injection induced a significant increase in circulating NK and CD8^+^ T cells with moderate toxicity.^196^ However, challenges persist in achieving sustained IL-15 exposure due to its short serum half-life, which restricts its immunostimulatory potency. The biostability of IL-15 is predominantly restricted by the availability of IL-15Rα. Consequently, various strategies have been employed to surmount these obstacles, involving the development of IL-15/IL-15Rα complexes or IL-15 superagonists.^197^

Notably, hetIL-15, which is designed based on the natural heterodimeric state of IL-15 and IL-15Rα for higher biostability, exhibits promising outcomes in preclinical models and ongoing clinical trials (Table 6).^198^ Its sustained plasma IL-15 levels and robust expansion of NK and T cells underscore its potential as a monotherapy for patients with metastatic or unresectable solid tumors.^199,200^ Likewise, hetIL-15Fc, a glycosylated form covalently linked to the Fc region of human IgG1, demonstrates superior efficacy in murine models.^201,202^ N-803, an IL-15 superagonist consisting of IL-15 variant fused with an IL-15Rα sushi domain and an Fc fragment, stands out with a remarkable half-life and increased bioactivity, showcasing its potential to eliminate established tumors and enhance NK cell cytotoxicity.^203^ Clinical trials further support the tolerability and efficacy of N-803, positioning it as a promising candidate for advanced cancer treatment.^204–208^ The continued exploration of IL-15 variants, including receptor-linker-IL-15 (RLI) and NKTR-255, further diversifies the therapeutic landscape, holding the potential to rescue NK cell activity and exhibit enhanced antitumor activity in various malignancies.^209–213^

**Table 6:** Clinical trials involving IL-15 for cancer therapy

Products	NCT number	Cancer types	Combination partners	Phase	Status
N-803	NCT03022825	Bladder Cancer	BCG	II/III	Recruiting
	NCT04847466	GEJC and HNSCC	Pembrolizumab and PD-L1 t-haNK	II	Recruiting
	NCT05445882	CRPC	M7824 and BN-Brachyury	II	Not yet recruiting
	NCT02138734	Bladder Cancer	BCG	I/II	Recruiting
	NCT06149481	Colorectal Cancer	SX-682, TriAdeno Vaccine, and Retifanlimab	I/II	Not yet recruiting
	NCT06253494	Endometrial Cancer	Pembrolizumab, Lenvatinib and HER2 Targeting Autologous Dendritic Cell (AdHER2DC) Vaccine	I/II	Not yet recruiting
	NCT05642195	NSCLC	Cancer Lysate Vaccine and Montanide ISA-51 VG	I/II	Recruiting
	NCT04491955	Colorectal Cancer	CV301, MSB0011359C, and NHS-IL12	II	Active, not recruiting
	NCT04247282	HNSCC	M7824 and TriAd vaccine	I/II	Completed
	NCT04927884	TNBC	PD-L1 t-haNK, Sacituzumab, and Cyclophosphamide	I/II	Terminated
	NCT05007769	NSCLC	Ramucirumab and Atezolizumab	II	Withdrawn
	NCT03493945	Prostate Cancer	BN-Brachyury Vaccine, M7824, and Epacadostat	I/II	Recruiting
	NCT03520686	NSCLC	Pembrolizumab and Chemotherapy	III	Active, not recruiting
	NCT06239220	HNSCC	PD-L1 t-haNK and Cetuximab	II	Not yet recruiting
	NCT04290546	HNSCC	CIML NK cell Infusion, Ipilimumab, and Cetuximab	I	Recruiting
	NCT04390399	Pancreatic Cancer	SBRT, Cyclophosphamide, Gemcitabine, Nab-paclitaxel, Aldoxorubicin, and PD-L1 t-haNK	II	Active, not recruiting
	NCT03228667	NSCLC	Anti-PD-1/PD-L1 + PD-L1 t-haNK	II	Active, not recruiting
	NCT02989844	AML	Monotherapy	II	Completed
	NCT06161545	HNSCC	Pembrolizumab and PD-L1 t-haNK Cells	II	Not yet recruiting
	NCT06061809	Glioblastoma	PD-L1 t-haNK and Bevacizumab	II	Not yet recruiting
	NCT05618925	Non-Hodgkin’s Lymphoma	CD19t-haNK suspension, Cyclophosphamide, Fludarabine, and Rituximab	I	Not yet recruiting
BJ-001	NCT04294576	Solid Tumors	Pembrolizumab	I	Active, not recruiting
NKTR-255	NCT05632809	Lung Cancer	Durvalumab	II	Recruiting
	NCT05676749	NSCLC	C-TIL051 and Pembrolizumab	I	Not yet recruiting
	NCT04616196	HNSCC	Cetuximab	I/II	Completed
	NCT03233854	B Acute Lymphoblastic Leukemia	Anti-CD19/CD22 CAR-T therapy	I	Recruiting
	NCT04136756	MM and Non-Hodgkin Lymphoma	Rituximab/Daratumumab	I	Completed
	NCT05327530	Urothelial Carcinoma	Avelumab	II	Recruiting
	NCT05664217	Non-Hodgkin Lymphoma and DLBL	Anti-CD19 CAR-T Therapy	II/III	Recruiting
	NCT05359211	DLBL	Anti-CD19 CAR-T Therapy	I	Recruiting

Note: *GEJC* gastroesophageal junction cancer, *CRPC* castration resistant prostate cancer, *TNBC* triple negative breast cancer, *AML* acute myelogenous leukemia, *MM* multiple myeloma, *DLBL* diffuse large B-cell lymphoma, *HNSCC* head and neck squamous cell carcinoma

Moreover, IL-15 is widely used to improve the efficacy of adoptive cell therapies against cancer, especially CAR-T cells.^214^ This novel approach involves not only ex vivo precultures but also the incorporation of IL-15 and its receptor within CAR engineering.^197^ IL-15-armored CAR-T cells have shown promising results, with enhanced expansion, prolonged persistence, and reduced cell death, leading to superior antitumor effects.^215,216^ Membrane-bound IL-15 (mbIL-15) signaling enhanced the persistence of T-memory stem cells and CAR-T cell efficacy.^217^ Clinical trials involving CAR T cells expressing transgenic mbIL-15 have demonstrated both effectiveness and safety, showcasing potential in treating hematological malignancies.^218,219^ Additionally, IL-15 or IL-15/IL-15Rα complex has been successfully integrated into NK cells, overcoming their short lifespan and improving NK cell survival.^220–223^ The application of IL-15 in unconventional T cells, such as invariant natural killer T (iNKT) and gamma delta T (γδT) cells, further extends its application, with IL-15-armed iNKT and γδT cells demonstrating enhanced proliferation ability and antitumor activity.^224,225^ Despite the encouraging outcomes, safety concerns have been raised, particularly in IL-15-armed NK cell therapy, emphasizing the need for careful evaluation and refinement of these innovative approaches in cancer immunotherapy.^220^ Moreover, emerging strategies like IL-15-armed oncolytic viruses and tumor-conditional IL-15 pro-cytokines offer the capability to induce localized expansion of NK cells and T cells with minimal systemic toxicity.^226–228^ These innovative approaches highlight the promising potential of IL-15-based therapies in reshaping the landscape of cancer immunotherapy.

### IL-1

#### IL-1 signaling and its protumor role

IL-1 is a potent DAMP, which was initially identified as a neutrophil-derived endogenous pyrogen.^229^ Subsequent investigations have elucidated its membership in a superfamily comprising 11 analogous molecules, each contributing to the intricate balance of pro-inflammatory and anti-inflammatory processes, particularly in the regulation of innate immune function.^230^ This family includes pro-inflammatory cytokines such as IL-1α, IL-1β, IL-18, IL-33, and IL-36α/β/γ, alongside anti-inflammatory counterparts like IL-1Ra, IL-33, IL-36Ra, IL-37, and IL-38.^230^ Notably, despite their significant homology and shared signaling redundancy, IL-1α and IL-1β exhibit distinct cellular origins, molecular regulations, and physiological roles in promoting inflammation.^231^ IL-1α serves as a paracrine DAMP, primarily released from cells undergoing severe physiologic stress or death, activating nearby cells to initiate a robust damage response.^232^ On the contrary, IL-1β functions as a systemic mediator of inflammation, triggered in response to distinct danger signals.^233^ IL-1α predominantly exerts its biological functions by binding to IL-1R1, a receptor featuring three primary ligands: IL-1α, IL-1β, and IL-1Ra.^234^ While IL-1α and IL-1β activate downstream signal transduction pathways, IL-1Ra acts as an endogenous inhibitor of IL-1R1 activity. Binding of either IL-1α or IL-1β to IL-1R1 initiates potent inflammation by canonical NF-κB and MAPK signaling pathways.^235^ This cascade involves the recruitment of IL-1RAcP, followed by the association of MYD88 and IRAK4.^236,237^ Subsequent autophosphorylation of IRAK4, phosphorylation of IRAK1/2, and the activation of TRAF6 trigger downstream signal transduction.^238–241^ TRAF6, an E3 ubiquitin ligase, forms K63-linked polyubiquitin chains crucial for activating NF-κB and MAPK pathways.^242^ As a results, the transcription of multiple IL-1-dependent pro-inflammatory mediators is upregulated, such as CXCL1/2, IL-6, and IL-8.^243^

IL-1 plays a multifaceted role in cancer, influencing various stages from carcinogenesis to metastasis. Elevated IL-1 levels are associated with poor prognosis in different cancers,^244^ and its production can be initiated by some oncogenic pathways, such as RAS signaling.^245^ IL-1 participates in carcinogenesis by promoting chronic inflammation and fostering a protumor cytokine network.^245,246^ It also mediates tumor angiogenesis by enhancing pro-angiogenic factor expression and endothelial cell activation.^247,248^ The involvement of IL-1 extends to therapy resistance, where it is linked to poor responses to EGFR tyrosine kinase inhibitor (TKI), radiotherapy, and other targeted therapies.^249–252^ Notably, the influences of IL-1 on antitumor immunity are paradoxical. While it exhibits antitumor effects by promoting the activation of NK and T cells, IL-1 contributes to cancer immunosuppression by improving the expansion and mobilization of immune cells such as MDSCs.^253–255^ These contradictory investigations underscore the pleiotropic nature of IL-1 signaling, confirming its dual impact in both promoting and suppressing tumors during cancer initiation and progression.^233^ Nevertheless, a substantial body of preclinical and clinical data overwhelmingly supports the notion that IL-1 predominantly operates in a protumor manner.^235^ Consequently, targeting IL-1 emerges as a potential therapeutic strategy, with ongoing clinical trials exploring the efficacy of anti-IL-1 therapies in various cancer types.

#### Anti-IL-1 therapy

At present, IL-1-based therapy has revealed promising avenues for therapeutic intervention in clinical trials. The strategies employed to target IL-1 signaling include direct inhibition of the IL-1 receptor, selective neutralization of IL-1α or IL-1β ligands with blocking antibodies, and targeted therapies against downstream molecules activated by the IL-1R1/MyD88 complex.^235^ Anakinra, a recombinant IL-1Ra, has secured FDA approval for rheumatoid arthritis and rare disorders.^256^ Beyond its established role in inflammatory diseases, anakinra has undergone small-scale clinical trials in solid tumors, exhibiting notable outcomes. Clinical studies using daily subcutaneous anakinra in patients with HER2-negative metastatic breast cancer demonstrated IL-1 receptor blockade-induced downregulation of genes involved in IL-1 and NF-κB signaling among circulating blood leukocytes.^257^ Additionally, anakinra in combination with standard chemotherapy and bevacizumab in metastatic colorectal cancer patients displayed well-tolerated results, with radiographic responses and stable disease observed.^258^ Notably, ongoing trials exploring isunakinra (an alternative form of rhIL-1Ra) plus anti-PD-1/L1 antibodies in solid tumors hold promise for further insights into IL-1Ra efficacy.^259^

Bermekimab/MABp1, an anti-IL-1α monoclonal antibody, has shown encouraging results in advanced colorectal cancer, as demonstrated in multiple clinical trials (Table 7).^260^ The phase I study exhibited a substantial reduction in serum IL-6 levels and an increase in lean body mass in patients, with notable responses observed, particularly in *KRAS*-mutant colon adenocarcinoma.^261^ Despite promising results, a phase III study, focusing on the improvement of quality-of-life metrics and lean body mass rather than traditional tumor-specific endpoints, showed some negative results.^262^ While patients treated with MABp1 demonstrated a significant improvement in the composite primary endpoint compared to placebo, post-hoc analysis revealed no significant improvements in individual quality-of-life scores with IL-1α neutralization.^262^ Furthermore, the termination of a subsequent phase III study (NCT01767857) due to treatment futility underscores the challenges of IL-1α inhibitor monotherapy in solid tumors, raising crucial questions about potential combinatorial treatment strategies in different clinical settings.^262^ Moreover, canakinumab, an anti-IL-1β monoclonal antibody, has emerged as a compelling therapeutic agent.^263^ The CANTOS trial demonstrated its efficacy in reducing cancer mortality (3.7 years post-treatment, hazard ratio [HR]: 0.49; *P* = 0.0009), particularly in lung cancer (canakinumab dose: 300 mg; HR: 0.23; *P* = 0.0002).^264^ Ongoing trials in advanced NSCLC explore canakinumab in combination with chemotherapy and immunotherapy, presenting a potential breakthrough in cancer treatment.^265,266^ These studies collectively underscore the intricate role of IL-1β blockade in impeding active disease progression and emphasize the need for further research into canakinumab efficacy as a pivotal element in IL-1-based cancer therapies.

**Table 7:** Clinical trials inhibiting IL-1 for cancer therapy

Products	NCT number	Cancer types	Combination partners	Phase	Status
Anakinra (IL-1 receptor antagonist)	NCT01802970	Breast Cancer	Chemotherapy	I	Completed
	NCT02090101	Colorectal Cancer	LV5FU2 and Bevacizumab	II	Completed
	NCT04942626	Rectal Cancer	Capecitabine-based Chemoradiotherapy	I	Active, not recruiting
	NCT00072111	Solid Tumors	Monotherapy	I	Completed
	NCT02021422	Pancreas Cancer	Oxaliplatin, Irinotecan, and Fluorouracil	I	Unknown status
	NCT01624766	Solid Tumors	Everolimus	I	Completed
	NCT00635154	MM	Dexamethasone	II	Completed
	NCT04227275	mCRPC	CART-PSMA-TGFβRDN genetically modified T cells, Cyclophosphamide, and Fludarabine	I	Terminated
	NCT02550327	Pancreatic Adenocarcinoma	Gemcitabine, Nab-Paclitaxel, and Cisplatin	I	Completed
	NCT03430011	MM	JCARH125	I/II	Completed
	NCT02492750	MM	Lenalidomide and Dexamethasone	I	Completed
	NCT04432506	B-Cell Lymphoma	Axicabtagene Ciloleucel, Cyclophosphamide, and Fludarabine	II	Active, not recruiting
	NCT04926467	Pancreatic Adenocarcinoma	Chemotherapy	II	Not yet recruiting
	NCT04150913	Non Hodgkin’s Lymphoma	Axicabtagene Ciloleucel	II	Active, not recruiting
	NCT04691765	Chronic Lymphocytic Leukemia	Monotherapy	I	Unknown status
	NCT04205838	DLBCL	Axicabtagene Ciloleucel, Cyclophosphamide, and Fludarabine	II	Recruiting
Canakinumab (Anti-IL-1β mAb)	NCT05725343	Lung Cancer	Monotherapy	III	Terminated
	NCT05984602	Pancreatic Cancer	Tislelizumab, Nab-Paclitaxel, and Gemcitabine	I	Recruiting
	NCT03447769	NSCLC	Monotherapy	III	Terminated
	NCT04905316	NSCLC	Chemotherapy, Radiation Therapy, and Durvalumab	II	Active, not recruiting
	NCT03968419	NSCLC	Pembrolizumab	II	Terminated
	NCT03631199	NSCLC	Pembrolizumab Plus Platinum-based Doublet Chemotherapy	III	Active, not recruiting
	NCT03626545	NSCLC	Docetaxel	III	Terminated
	NCT03742349	TNBC	Spartalizumab and LAG525	I	Terminated
	NCT02900664	Colorectal Cancer, TNBC, and NSCLC	Spartalizumab	I	Completed
	NCT04229004	Pancreatic Adenocarcinoma	Spartalizumab, Nab-paclitaxel, and Gemcitabine	III	Active, not recruiting
	NCT04581343	Pancreatic Ductal Adenocarcinoma	Spartalizumab, Nab-paclitaxel, and Gemcitabine	I	Active, not recruiting
	NCT03064854	NSCLC	Spartalizumab Plus Platinum-doublet Chemotherapy	I	Terminated
	NCT04028245	ccRCC	Spartalizumab	I	Recruiting
	NCT03484923	Melanoma	Spartalizumab	II	Completed
MABp1 (Anti-IL-1α mAb)	NCT01021072	Solid Tumors	Monotherapy	I	Completed
	NCT01767857	Colorectal Cancer	Monotherapy	III	Terminated

Note: *MM* multiple myeloma, *mCRPC* metastatic castration-resistant prostate cancer, *DLBCL* diffuse large B-cell lymphoma, *NSCLC* non-small cell lung cancer, *TNBC* triple negative breast cancer, *ccRCC* clear cell renal cell carcinoma

### IL-6

#### The role of IL-6 signaling in cancer progression and immune-related adverse events

IL-6 is a multifaceted cytokine playing critical roles in immune responses, inflammation, and a range of physiological processes such as hematopoiesis, bone metabolism, and embryonic development.^267^ Its significance is particularly noted in the pathophysiology of various diseases, including cancer.^268^ IL-6 signals through three distinct pathways: classical, trans-signaling, and trans-presentation signaling.^269^ Classical signaling involves IL-6 binding to its membrane-bound receptor (mIL-6R), leading to gp130 receptor dimerization and signal transduction.^270^ Trans-signaling allows cells without mIL-6R to respond to IL-6 via the soluble form of IL-6R (sIL-6R).^271^ Trans-presentation signaling facilitates IL-6 presentation from mIL-6R on one cell to gp130 on another, broadening cellular responses.^272^ Classical signaling is crucial for acute-phase immune responses, hematopoiesis, and homeostasis.^273^ Trans-signaling plays a vital role in the TME by modulating immune cell recruitment and stromal cell inflammatory responses.^273^ Trans-presentation signaling is essential for pathogenic Th17 cell priming.^272^

The dysregulation of IL-6 signaling, particularly via the JAK-STAT3 pathway, has been identified as a pivotal contributor to tumorigenesis.^274^ The JAK-STAT3 pathway is initiated by the formation of hexameric IL-6/IL-6Rα/gp130 complex, subsequently ensuing in gp130 phosphorylation and STAT3 activation.^275^ The activated STAT3 then migrates to the nucleus, where it modulates gene expression related to cell cycle progression, survival, and angiogenesis, including cyclin-D1, Bcl-2, c-Myc, Bcl-xL, survivin, VEGF, MMP-2, and IL-6 itself.^276–285^ Importantly, this signaling pathway not only directly fosters tumor growth but also significantly contributes to immune evasion by altering the TME.^286^ IL-6 undermines immune surveillance by regulating the immunosuppressive capacity of MDSCs, inhibiting antigen presentation, and upregulating immune checkpoint molecules.^287–290^ Consequently, IL-6-mediated immune suppression diminishes the efficacy of ICB therapies, with IL-6 levels serving as predictive markers for ICB response.^291,292^ Preclinical investigations have shown that IL-6 inhibition, in synergy with ICB, amplifies antitumor immunity and curtails tumor progression across various cancer models.^293^ Additionally, IL-6 has been implicated in intensifying immune-related adverse events (irAEs) associated with ICB, suggesting its significant impact on patient management beyond mere tumor suppression.^294^ The strategic combination of IL-6 targeting agents with ICB not only holds promise for augmenting cancer treatment efficacy but also for managing irAEs, as demonstrated by the effective application of the anti-IL-6R antibody tocilizumab in clinical practice.^295^

#### IL-6 blockade to improve immunotherapy efficacy and mitigate adverse events

Therapeutic approaches to inhibit IL-6 signaling are principally divided into two main categories: antibodies targeting IL-6 or its receptor, and small-molecule inhibitors of JAK and STAT3. In addition to these conventional strategies, innovative blockade techniques have emerged, including the development of sgp130-Fc fusion proteins, STAT3 antisense oligonucleotides, and cyclic STAT3 decoys.^296^ These novel approaches offer alternative mechanisms to modulate the IL-6 signaling axis, potentially overcoming the limitations of existing therapies and providing new avenues for the treatment of diseases mediated by aberrant IL-6 signaling.

Anti-IL-6/IL-6R monoclonal antibodies such as tocilizumab, sarilumab, and siltuximab, initially approved for indications like rheumatoid arthritis and Castleman disease, have been repurposed with promising implications for cancer, particularly in managing cytokine release syndrome associated with CAR-T cell therapy (Table 8).^297^ The development of novel blockade strategies, including sgp130-Fc fusion proteins, has expanded the therapeutic arsenal, aiming to selectively inhibit IL-6 trans-signaling without compromising immune defense mechanisms.^298–300^ Despite the therapeutic potential, challenges such as increased risk of bacterial infections and limited efficacy in unselected patient populations highlight the complexity of targeting IL-6 in cancer. Clinical trials investigating the antitumor efficacy of siltuximab have shown mixed results, underscoring the necessity for combination therapies and the identification of predictive biomarkers to enhance treatment outcomes.^301–303^ The exploration of tocilizumab in various cancers through early-phase trials further exemplifies ongoing efforts to harness anti-IL-6 strategies, potentially offering new avenues for cancer therapy by mitigating pro-inflammatory effects while preserving immune surveillance.^304,305^

**Table 8:** Clinical trials of IL-6 blocking antibodies for cancer therapy

Products	NCT number	Cancer types	Combination partners	Phase	Status
Siltuximab	NCT00311545	Kidney Cancer	Monotherapy	II	Withdrawn
	NCT00433446	Prostate Cancer	Monotherapy	II	Completed
	NCT00385827	Prostate Cancer	Mitoxantrone and Prednison	II	Terminated
	NCT04191421	Pancreatic Cancer	Spartalizumab	I/II	Completed
	NCT00401765	Prostate Cancer	Docetaxel	I	Completed
	NCT00841191	Solid Tumors	Monotherapy	I/II	Completed
	NCT01309412	MM	Monotherapy	I	Terminated
	NCT00402181	MM	Dexamethasone	II	Completed
	NCT01266811	MM	Velcade and Dexamethasone	III	Withdrawn
	NCT00401843	MM	Bortezomib and Dexamethasone	II	Completed
	NCT01531998	MM	Lenalidomide, Bortezomib, and Dexamethasone	I/II	Completed
	NCT00911859	MM	Velcade, Melphalan, and Prednisone	II	Completed
	NCT01484275	MM	Monotherapy	II	Completed
	NCT05697510	AML	Monotherapy	I	Recruiting
	NCT00265135	RCC	Monotherapy	I/II	Completed
	NCT00412321	Non-Hodgkin’s Lymphoma and MM	Monotherapy	I	Completed
	NCT05316116	LGLL	Monotherapy	I	Recruiting
	NCT05665725	Non-Hodgkin’s Lymphoma	Monotherapy	I	Recruiting
Tocilizumab	NCT06016179	Metastatic Cancer	Monotherapy	I	Recruiting
	NCT05846789	Breast Cancer	Carboplatin	II	Recruiting
	NCT05619744	SCLC and Neuroendocrine Carcinoma	RO7616789	I	Recruiting
	NCT05129280	Solid Tumors	RO7444973	I	Terminated
	NCT04940299	Melanoma, NSCLC, or Urothelial Carcinoma	Ipilimumab and Nivolumab	II	Active, not recruiting
	NCT04691817	NSCLC	Atezolizumab	I/II	Recruiting
	NCT04547062	AML	Monotherapy	I	Completed
	NCT04375228	Solid Tumors	Monotherapy	II	Recruiting
	NCT04338685	Hepatocellular Carcinoma, Biliary Tract Cancer, Or Tumors with Hepatic Metastases	RO7119929	I	Completed
	NCT04258150	Pancreatic Cancer	Nivolumab, Ipilimumab, and SBRT	II	Terminated
	NCT03999749	Melanoma	Ipilimumab and Nivolumab	II	Active, not recruiting
	NCT03866239	Colorectal Cancer	Obinutuzumab, Atezolizumab, and Cibisatamab	I	Active, not recruiting
	NCT03821246	Prostate Cancer	Atezolizumab and Etrumadenant	II	Recruiting
	NCT03708224	HNSCC	Atezolizumab and Tiragolumab	II	Recruiting
	NCT03588936	Hematological Malignancy	Nivolumab	I	Terminated
	NCT03424005	Breast Cancer	Atezolizumab and Nab-Paclitaxel	I/II	Recruiting
	NCT03135171	Breast Cancer	Trastuzumab and Pertuzumab	I	Completed
	NCT02997956	Hepatocellular Carcinoma	Transcatheter Arterial Chemoembolization	I/II	Withdrawn
	NCT02906371	Lymphoblastic Leukemia	CART19 Therapy	I	Completed
	NCT02767557	Pancreatic Carcinoma	Nab-Paclitaxel and Gemcitabine	II	Completed
	NCT01637532	Ovarian Cancer	Chemotherapy and Peg-Intron	I/II	Completed
Sarilumab	NCT05704634	NSCLC	Cemiplimab	I	Recruiting
	NCT04333706	TNBC	Capecitabine	I/II	Recruiting
	NCT03972657	mCRPC and ccRCC	REGN5678 and Cemiplimab	I/II	Recruiting
	NCT03564340	Ovarian Cancer or Other MUC16+ Cancers	REGN4018	I/II	Recruiting
	NCT05125016	mCRPC	REGN4336 and Cemiplimab/REGN5678	I/II	Recruiting
	NCT05428007	Melanoma	Ipilimumab and Nivolumab/Relatlimab	II	Recruiting

Note: *AML* acute myeloid leukemia, *RCC* renal cell carcinoma, *LGLL* large granular lymphocytic leukemia, *mCRPC* metastatic castration-resistant prostate cancer, *ccRCC* clear cell renal cell carcinoma, *NSCLC* non-small cell lung cancer, *SCLC* small cell lung cancer, *MM* multiple myeloma

Besides, small-molecule inhibitors targeting downstream elements of the IL-6 signaling pathway, such as JAK and STAT3, show promise in cancer treatment as well. JAK inhibitors, such as tofacitinib and ruxolitinib, have been approved for various inflammatory diseases and myeloproliferative neoplasms, demonstrating their potential to modulate immune responses.^306,307^ Despite preclinical data suggesting JAK inhibitors could retard solid tumor growth, clinical evidence supporting their use in solid tumors is limited.^308^ At present, ongoing early-phase trials continue to evaluate the safety and potential efficacy of JAK inhibitors in various solid cancers, aiming to identify therapeutic windows that balance efficacy with tolerability.^309,310^ For instance, antisense oligonucleotides like AZD9150 have shown activity against treatment-refractory lymphoma and NSCLC, with a maximum-tolerated dose established at 3 mg/kg, showcasing a favorable safety profile.^311^ Moreover, early-phase clinical trials for nonpeptide SH2 domain antagonists such as OPB-31121 and OPB-51602 have provided evidence of antitumor activity, particularly in hepatocellular carcinoma and NSCLC, despite facing tolerability challenges like peripheral neuropathy and pneumonitis.^312–315^

Notably, integrating anti-IL-6 therapies with ICB represents a promising approach to overcoming immunosuppression driven by cancer-promoting inflammation. The complexity of chronic inflammation, regulated by numerous pathways and compensatory mechanisms, has limited the efficacy of cytokine-targeting drugs as monotherapies. However, robust preclinical evidence supports the combination of IL-6 signaling blockade with ICB as an attractive strategy for enhancing treatment efficacy in solid tumors, potentially boosting ICB effectiveness and mitigating irAEs.^294,316^ The efficacy of tocilizumab in treating ICB-induced colitis and arthritis was evaluated in the COLAR study.^317^ Nineteen patients received tocilizumab treatment (8 mg/kg) every four weeks until symptoms worsened or unacceptable toxicity, without the use of systemic glucocorticoids or other immunosuppressive drugs within a 14-day follow-up period.^317^ The primary endpoint, clinical improvement in colitis and arthritis, specifically achieving a reduction of at least one grade in the CTCAE within an 8-week period, was achieved by 79% of the patients, with ongoing improvement or complete remission in 12 patients at week 24, without the need for glucocorticoids. The trial supports the feasibility of randomized trials for tocilizumab as a treatment for ICB-induced colitis and arthritis.^317^ Additionally, the use of JAK and STAT3 inhibitors combined with ICB in advanced cancers, exemplified by ruxolitinib-alleviated ICB-associated myocarditis, underscores the potential of targeting the IL-6/JAK/STAT3 signaling pathway to augment antitumor immunity and address the adverse inflammatory effects of ICB treatment.^318,319^ This evolving paradigm suggests a synergistic potential that could redefine treatment strategies for patients with advanced-stage cancers.

## TNF signaling and TNF blockade for immunotherapy

### TNF signaling: from direct tumoricidal effects to multifaceted protumor activities

TNF was first isolated as a crucial factor responsible for endotoxin-induced hemorrhagic necrosis of tumors.^320^ The cloning of the *TNF* gene in the 1980s expanded the understanding of its role, revealing its identity as cachectin, a key player in the physiological responses to infection, including acute shock and chronic cachexia.^321^ Subsequent research highlighted the complex role of TNF in cancer, initially seen as a promising anti-cancer agent due to its ability to induce tumor necrosis.^322^ However, its potential as a therapeutic has been limited by a narrow therapeutic window. At physiologically tolerable levels, TNF alone is not directly cytotoxic to cancer cells.^323^ Currently, our understanding of the biological functions of TNF has undergone significant evolution. Beyond its direct tumoricidal effects under specific conditions, TNF has been implicated in promoting tumor progression. The protumor activities of TNF are multifaceted, involving the modulation of the TME to favor cancer cell proliferation, survival, and metastasis.^324^ This includes the induction of angiogenesis, a process crucial for tumor growth and metastasis, whereby TNF stimulates the formation of new blood vessels, ensuring a steady supply of nutrients and oxygen to rapidly growing tumors.^325^

Furthermore, TNF has been shown to contribute to cancer immune evasion. Preclinical studies have revealed that TNF hinders the accumulation of CD8^+^ T cells in tumor-draining lymph nodes and tumors through TNFR-mediated activation-induced cell death (AICD) in CD8^+^ T cells.^326^ Moreover, TNF undermines the antitumor activity of NK cells by upregulating TIM-3 and downregulating NKp46.^327,328^ Furthermore, TNF promotes Treg proliferation and suppressive functions, which in turn dampens the overall immune response against tumors. This effect is particularly pronounced in Treg cells that express TNFR2, which are found in high densities within the TME and contribute to tumor growth by suppressing non-Treg cell proliferation.^329,330^ Conversely, TNF enhances Th cell proliferation and pro-inflammatory cytokine production, but this effect is complicated by TNF inhibitors potentially promoting Th1 cell function indirectly by restraining Treg cells.^331^ TNF also plays a role in the survival and immunosuppressive activity of MDSCs.^332,333^ Additionally, TNF stimulates mesenchymal stem cells (MSCs) to recruit CCR2-positive tumor-associated macrophages (TAMs) into the TME, further supporting tumor growth.^334^ Also, TNF increases PD-L1 surface expression on cancer cells by stabilization of PD-L1.^335^ Therefore, inhibiting TNF presents a promising strategy not only to enhance the antitumor immune response by improving T cell and NK cell function and restraining immunosuppressive Treg, MDSCs, and MSCs but also to directly inhibit cancer cell survival and proliferation, illustrating the multifaceted role of TNF in cancer immunology and the potential benefits of its inhibition. Preclinical studies have demonstrated that TNF blockade enhances the therapeutic effect of anti-PD-1 treatment, elevating tumor rejection rates from 20% with anti-PD-1 alone to 75% when combined with TNF inhibition.^336,337^

### TNF blockade to improve immunotherapy efficacy and alleviate adverse events

In addition to synergistic antitumor effects, of greater interest is the value of TNF blockade in mitigating irAEs, especially IBD-induced colitis. Elevated TNF levels were found in patients with colitis after treatment with ipilimumab and nivolumab. In the xenograft model, preventive TNF blockade not only alleviates colitis and hepatitis in the mice but also maintains the efficacy of immunotherapy.^338^ Actually, anti-TNF antibodies such as infliximab and adalimumab have been widely used for the treatment of inflammatory bowel disease and some autoimmune diseases such as rheumatoid arthritis.^339,340^ Badran et al. reported five cancer patients treated with ICB developed immune-related enterocolitis (irEC) within 40 days of treatment onset, confirmed by endoscopy to be acute inflammation. Initial treatment with steroids was supplemented by adding infliximab to avoid long-term steroid use and gastrointestinal symptom recurrence. This combination therapy allowed continued ICB treatment, with follow-up checks showing inflammation resolution and no cancer progression. This suggests that combining anti-TNF-α with ICB is a promising strategy for safely managing irEC.^341^ Moreover, the TICIMEL phase Ib clinical trial (NTC03293784) evaluated the combination of TNF blockers (infliximab or certolizumab) with ICB in 14 advanced melanoma patients (Table 9).^342^ This trial aimed to assess the safety and antitumor efficacy of these combinations, with a particular focus on managing gastrointestinal side effects. The trial found both combinations to be safe, with only one dose-limiting toxicity reported in the infliximab group and generally lower treatment-related adverse events for infliximab compared to certolizumab.^342^ The certolizumab cohort had a notable response rate: 7 of 7 evaluable patients showed an objective response, including four complete responses. In contrast, the infliximab cohort recorded one complete response, two partial responses, and three progressive diseases. The results suggest the safety and potential antitumor benefits of these combinations.^342^

**Table 9:** Clinical trials involving TNF antagonist for cancer therapy

Products	NCT number	Cancer types	Combination partners	Phase	Status
Infliximab	NCT05034536	Melanoma	Pembrolizumab	II	Recruiting
	NCT04407247	Genitourinary Cancer or Melanoma	Monotherapy	I/II	Recruiting
	NCT04305145	Melanoma	Monotherapy	II	Unknown status
	NCT04082910	Solid and Hematological Malignancy	Metoprolol	I/II	Recruiting
	NCT03293784	Melanoma	Nivolumab and Ipilimumab	I	Completed
	NCT02763761	RCC, Melanoma, and Lung Cancer	Monotherapy	II	Withdrawn
	NCT00112749	Breast Cancer	Monotherapy	II	Terminated
	NCT00060502	Pancreatic Neoplasms	Gemcitabine	II	Completed
	NCT00040885	Lung Cancer	Docetaxel	III	Completed
Etanercept	NCT00201812	Solid Tumors	Docetaxel and Dexamethasone	I	Completed
	NCT00046904	Solid Tumors	Monotherapy	III	Completed
	NCT00201838	Pancreatic Neoplasms	Gemcitabine	I/II	Completed
	NCT03792841	Prostate Cancer	Acapatamab	I	Completed
	NCT00127387	Solid Tumors	Monotherapy	II/III	Terminated
	NCT04082910	Solid and Hematological Malignancy	Metoprolol	I/II	Recruiting
Adalimumab	NCT02516774	Anaplastic Thyroid Cancers	Monotherapy	I	Withdrawn
Golimumab	NCT05960578	Prostate Cancer	Apalutamide	II	Recruiting

Note: *RCC* renal cell carcinoma

## Utilizing chemokines in cancer therapy

**Fig. 6:**
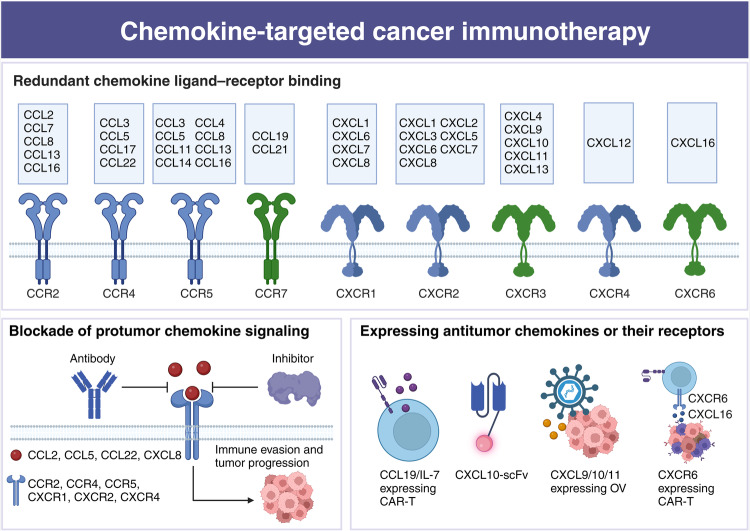
Chemokine-targeted cancer immunotherapy. The diagram presents the complexity of chemokine ligand-receptor interactions and their implications for cancer immunotherapy. The top section identifies the chemokine ligands (e.g., CCL2, CCL7, CXCL9) and their corresponding receptors, categorized by their role in tumor progression, with antitumor receptors labeled in green (e.g., CXCR3, CXCR6) and protumor receptors in blue (e.g., CCR2, CXCR1, CXCR2). The bottom left panel highlights the blockade of protumor chemokine signaling using antibodies and inhibitors targeting specific CCL and CXCL chemokines and their receptors to prevent immune evasion and tumor progression. The bottom right panel showcases the expression of antitumor chemokines or their receptors, such as CCL19/IL-7 expressing CAR-T cells, CXCL10-scFv, CXCL9/CXCL10/CXCL11 expressing oncolytic viruses (OVs), and CXCR6 expressing CAR-T cells, as innovative strategies to enhance antitumor immunity. This figure encapsulates the dual approach of inhibiting tumor-promoting chemokines and augmenting antitumor chemokines to therapeutically modulate the tumor microenvironment. (Created with BioRender.com)

The role of chemokines in cancer involves a complex interplay among cancer cells, tissue-resident cells, and immune cells. These chemokines influence tumor cell behavior by affecting their stemness, proliferation, and invasiveness, as well as impacting stromal cells to modulate processes like angiogenesis and fibrogenesis.^343^ Importantly, chemokines also shape the phenotype and function of immune cells within both lymphoid tissues and the TME. On the one hand, they orchestrate the recruitment and spatial organization of immune cells, facilitating their interactions within tissues, which is crucial for triggering antitumor immune response. On the other hand, chemokines also contribute to the formation of protumor microenvironment.^343^ The balance between antitumor and protumor roles of chemokines depends on tumorigenesis stages, immune cell activation states, and the specific chemokine receptors expressed on target cells. Targeting chemokines that facilitate antitumor immune cell recruitment, or inhibiting those that enhance the suppressive immune cell function, presents promising strategies to enhance the efficacy of cancer therapies (Fig. 6).^344^

### CCL2-CCR2 axis

#### The protumor role of CCL2-CCR2 axis

The CCL2-CCR2 signaling axis plays a pivotal role in tumorigenesis, promoting the initiation, progression, and metastasis of various malignancies, including breast, lung, hepatocellular, gastric, esophageal, prostate, ovarian, and bladder cancers.^345–352^ It supports tumor growth and proliferation at the primary site and facilitates tumor metastasis.^353^ Moreover, CCL2-CCR2 signaling orchestrates an immunosuppressive TME by recruiting MDSCs, Tregs, TAMs, and other immune cells.^354–356^ This axis also significantly contributes to tumor angiogenesis by directly stimulating vascular endothelial cells and indirectly through the recruitment of inflammatory cells that express angiogenic factors.^357–359^ In addition to its role in recruiting immunosuppressive cell types, tumor-derived CCL2 impacts the function of effector T cells.^360^ Targeting the CCL2-CCR2 axis has emerged as a potential therapeutic strategy, aiming to inhibit the recruitment of protumor immune cells and disrupt the protumor TME, thus opening new avenues for cancer therapy.

#### CCL2-CCR2 axis blockade for cancer therapy

Agents targeting the CCL2-CCR2 axis have demonstrated promising antitumor activity in preclinical studies. Inhibition of CCL2, using various inhibitors or antibodies like C1142, bindarit, and curcumin, has been shown to suppress tumor growth by blocking CCL2-mediated signaling pathways, reducing immunosuppressive cell recruitment, and increasing effector T cell numbers.^361–363^ Similarly, targeting CCR2 with antagonists such as RS-504393 and RS-102895 has been effective in delaying tumor progression by inhibiting the infiltration of immunosuppressive cells into tumors.^364–366^ Moreover, combined therapy approaches, integrating CCL2-CCR2 axis blockade with existing cancer treatments, have been explored to overcome the complexity of cancer pathogenesis and minimize side effects. For instance, dual targeting of CCL2/CCR2 and PD-1 has yielded notable tumor suppression and improved survival of tumor-bearing mice.^367–369^ These advances underscore the importance of the CCL2-CCR2 axis in cancer immunology and its potential as a therapeutic target.

Encouraged by the positive results of preclinical studies, the antitumor activity and safety profile of CCL2/CCR2 antagonists have been intensively explored in clinical trials, particularly with agents such as Carlumab and PF-04136309 (Table 10). Carlumab, a human anti-CCL2 antibody, was well-tolerated in a phase I study involving patients with advanced solid tumors, showing no dose-limiting toxicity.^370^ However, its therapeutic impact was modest, with stable disease observed in a minority of patients but without any achieving an objective response.^370^ In a phase II study for metastatic castration-resistant prostate cancer, Carlumab did not lead to any prostate-specific antigen (PSA) response, and only 34% of patients maintained stable disease beyond three months.^371^ PF-04136309, a CCR2 inhibitor, exhibited promising antitumor activity in a phase Ib study when combined with FOLFIRINOX chemotherapy for pancreatic cancer, achieving tumor control in 97% of patients and objective tumor response in 49%.^372^ Moreover, CCX872-B, another CCR2 antagonist, combined with FOLFIRINOX for pancreatic adenocarcinoma, showed an 18-month OS rate of 29%, better than historical data of FOLFIRINOX regimen alone, suggesting a potential survival benefit.^373^ Notably, the study of PF-04136309 reported treatment-related serious adverse events in 66.7% of patients, especially synergistic pulmonary toxicity when combined with nab-paclitaxel/gemcitabine,^374^ highlighting the need for careful consideration of safety alongside therapeutic benefits.

**Table 10:** Clinical trials involving CCL2/CCR2 inhibitors for cancer therapy

Classification	Products	NCT number	Cancer types	Combination partners	Phase	Status
CCR2/5i	BMS-813160	NCT04123379	NSCLC and HCC	Nivolumab	II	Active, not recruiting
		NCT03184870	Colorectal and Pancreatic Cancer	Chemotherapy or Nivolumab	I/II	Completed
		NCT02996110	RCC	Nivolumab	II	Completed
		NCT03767582	Pancreatic Ductal Adenocarcinoma	SBRT, Nivolumab, and GVAX	I/II	Recruiting
		NCT03496662	Pancreatic Ductal Adenocarcinoma	Nivolumab, Gemcitabine, and Nab-paclitaxel	I/II	Active, not recruiting
CCR2i	CCX872-B	NCT03778879	Pancreatic Cancer	SBRT	I/II	Withdrawn
		NCT02345408	Pancreatic Cancer	Monotherapy	I	Completed
	PF-04136309	NCT01413022	Pancreatic Cancer	Oxaliplatin, Irinotecan, Leucovorin, and Fluorouracil	I	Completed
		NCT02732938	Pancreatic Ductal Adenocarcinoma	Nab-paclitaxel and Gemcitabine	II	Terminated
Anti-CCR2 mAb	MLN1202	NCT01015560	Solid Tumors	Monotherapy	II	Completed
		NCT02723006	Melanoma	Nivolumab	I	Terminated
Anti-CCL2 mAb	Carlumab	NCT00992186	Prostate Cancer	Monotherapy	II	Completed

Note: *NSCLC* non-small cell lung cancer, *HCC* hepatocellular carcinoma, *SBRT* stereotactic body radiation therapy, *RCC* renal cell carcinoma, *mAb* monoclonal antibody

### CCR4 signaling pathway

#### CCR4 signaling-mediated cancer immune evasion

The CCR4 signaling pathway plays a pivotal role in the TME, primarily through its expression on a majority of human Tregs (>90%).^375^ In various cancers, tumor cells, TAMs, and DCs secrete high levels of CCR4 ligands, CCL17 and CCL22, which facilitate Treg infiltration into tumor sites.^376,377^ This infiltration, driven by the interaction between CCR4 on Tregs and ligands produced by the tumor, has been correlated with a poor prognosis.^378^ The strategic blockade of this pathway, either through targeting CCL22 with monoclonal antibodies to reduce Treg migration into tumors or by directly inhibiting CCR4 to prevent its interaction with multiple chemokines, has shown promise.^379^ Direct CCR4 blockade, has demonstrated its efficacy by not only reducing Treg infiltration but also inhibiting tumor growth in xenograft mouse models, indicating the potential of CCR4 as a therapeutic target in cancer treatment.^380,381^ Apart from Tregs, CCR4 blockade regulates the TAM phenotype and decreases the presence of immature myeloid cells in the TME.^382,383^ Furthermore, CCR4-dependent Treg accumulation is a core factor contributing to ICB resistance. In Pan02 and CT26 mouse tumor models, CCR4 blockade decreases Treg migration, thereby improving ICB performance, particularly in tumors with high baseline CCR4 ligand expression or in those where ICB treatment upregulates CCR4 ligands.^384^ Consequently, inhibiting CCR4 not only reduces Treg frequency but also amplifies the efficacy of ICB, highlighting the importance of CCR4-dependent Treg recruitment in immunotherapy resistance and supporting the use of CCR4 inhibitors alongside ICB in cancer treatment strategies.^384^

#### CCR4 antagonists improving immunotherapy effectiveness especially ICB

At present, several CCR4 antagonists have undergone evaluation in clinical trials, however, mogamulizumab is the sole CCR4 antagonist approved for cancer treatment, specifically for treating T cell lymphomas.^385^ Besides, mogamulizumab effectively induced depletion of FoxP3^+^ Tregs in patients with solid tumors.^386^ In a phase I clinical trial, mogamulizumab was safe and well-tolerated, without any dose-limiting toxicity (Table 11). Notably, four out of ten patients exhibited stable disease and were categorized as long survivors. Treatment resulted in effective Tregs depletion at even the lowest dose, with minimal impact on Th1 T cells but significant reductions in Th2 and Th17 CD4^+^ T cells.^386^ Then in the multicenter phase I study (NCT02301130), the safety, antitumor efficacy, and pharmacodynamics of mogamulizumab combined with ICB (durvalumab or tremelimumab) were evaluated in patients with advanced solid tumors. No dose-limiting toxicities were reported across the 64 participants, and the treatment was found to be tolerable.^387^ However, the ORR stood at a mere 5.3%, indicating limited antitumor efficacy despite the effective depletion of peripheral and intratumoral Tregs by mogamulizumab. There was also no apparent correlation between the clinical response and the reduction in CCR4^+^ Tregs or baseline CCR4 expression.^387^ On the contrary, in another phase I clinical study NCT02476123, the combination of mogamulizumab and anti-PD-1 antibody nivolumab exhibited an acceptable safety profile and meaningful antitumor activity in solid tumors. In this trial, no dose-limiting toxicities were observed in the dose-escalation part.^388^ Grade 3/4 treatment-related adverse events occurred in 29% of patients in the expansion part. Besides, 27% of hepatocellular carcinoma patients (4 out of 15) showed confirmed tumor responses, and in the pancreatic adenocarcinoma cohort, there was one confirmed and two unconfirmed responses among 15 patients.^388^ This regimen also led to decreased populations of effector Tregs and increased CD8^+^ T cells within the TME.^388^ The discrepancy between the two trials underscores the critical role of the tumor microenvironment and the specific mechanisms of action of the therapeutic agents used. It suggests that the success of combining Treg depletion with ICB may be contingent on selecting the right combination of therapeutic agents, the cancer type, and understanding the underlying tumor immunobiology. Furthermore, these trials highlight the need for biomarker-driven patient selection and personalized approaches to immunotherapy. Identifying patients who are more likely to benefit from Treg depletion in combination with checkpoint inhibition could enhance the efficacy of such treatments and provide valuable insights into optimizing cancer immunotherapy strategies.

**Table 11:** Clinical trials involving Anti-CCR4 antibody mogamulizumab for cancer therapy

NCT number	Cancer types	Combination partners	Phase	Status
NCT02358473	NSCLC	Docetaxel	I	Completed
NCT02867007	Solid Tumors	KHK2455	I	Completed
NCT02946671	Solid Tumors	Nivolumab	I	Completed
NCT02281409	Solid Tumors	Monotherapy	I/II	Completed
NCT02301130	Solid Tumors	Durvalumab/Tremelimumab	I	Completed
NCT02476123	Solid Tumors	Nivolumab	I	Completed
NCT02444793	Solid Tumors	PF-05082566	I	Terminated
NCT01929486	Solid Tumors	Monotherapy	I	Unknown status
NCT02705105	Solid Tumors	Nivolumab	I/II	Completed
NCT01611142	T-Cell Lymphoma	Monotherapy	II	Completed
NCT04745234	T-Cell Lymphoma	Monotherapy	II	Active, not recruiting
NCT04128072	T-Cell Lymphoma	Total Skin Electron Beam Therapy	II	Recruiting
NCT05996185	T-Cell Lymphoma	DA-EPOCH Chemotherapy	II	Not yet recruiting
NCT00920790	T-cell Leukemia/lymphoma	Monotherapy	II	Completed
NCT03309878	DLBCL	Pembrolizumab	I/II	Completed
NCT01728805	T-Cell Lymphoma	Monotherapy	III	Completed
NCT05414500	T-Cell Lymphoma	Brentuximab vedotin	I	Recruiting
NCT05956041	T-Cell Lymphoma	Pembrolizumab	II	Recruiting
NCT04185220	T-Cell Lymphoma	Recombinant Human IL-15	I	Completed
NCT04930653	T-Cell Lymphoma	Extracorporeal Photopheresis	II	Recruiting
NCT04676087	Non-Hodgkin’s Lymphoma	Extracorporeal Photopheresis	I/II	Recruiting
NCT01226472	T-Cell Lymphoma	Monotherapy	II	Completed
NCT01192984	T/NK-cell Lymphoma	Monotherapy	II	Completed
NCT04848064	Lymphoma	NK cell infusion and Chemotherapy	I	Recruiting
NCT00355472	T-Cell Lymphoma	Monotherapy	I	Completed
NCT01173887	T-Cell Lymphoma	VCAP/AMP/VECP(mLSG15) Chemotherapy Strategy	II	Completed
NCT01626664	T-Cell Lymphoma	Monotherapy	II	Completed
NCT00888927	T-Cell Lymphoma	Monotherapy	I/II	Completed

Note: *NSCLC* non-small cell lung cancer, *DLBCL* diffuse large B cell lymphoma

### CCL5/CCR5 signaling pathway

#### CCL5/CCR5 signaling supporting tumor development

The CCL5/CCR5 signaling pathway plays a pivotal role in cancer development and progression.^389^ CCL5, also known as RANTES, is a chemokine primarily expressed by inflammatory cells, notably T cells and monocytes.^390^ It binds with the highest affinity to CCR5, a G-protein-coupled receptor (GPCR) found in various cell types, including T cells, smooth muscle, epithelial, and endothelial cells.^391^ The CCL5/CCR5 axis is involved in numerous physiological and pathological processes, such as HIV infection, cell proliferation, migration, angiogenesis, metastasis, and survival, making it a focal point of study in inflammation, cancer, and viral infections.^392,393^ The signaling pathways activated downstream of CCL5/CCR5 signaling, such as PI3K/AKT, MAPK, JAK-STAT, NF-κB, HIF-1α, and TGF-β-Smad, are implicated in promoting uncontrolled tumor cell proliferation, angiogenesis, apoptosis resistance, invasion, and metastasis.^392^ Recent research highlights the significant role of CCL5/CCR5 signaling in creating a protumor TME by recruiting Tregs, MDSCs, and TAMs, thereby contributing to tumor immunosuppression.^394–396^

#### CCL5/CCR5 blockade: from HIV infection treatment to cancer therapy

The CCL5/CCR5 axis has been identified as a target for therapeutic intervention, especially cancers like breast cancer.^397^ Current strategies focus on developing small molecule inhibitors like maraviroc, cenicriviroc, anibamine, vicriviroc, and MET-CCL5, which have shown potential in clinical evaluations for their anti-inflammatory and anti-cancer properties (Table 12).^398–402^ Maraviroc, an FDA-approved drug for HIV infection, repurposed in cancer therapy, competes with CCL5 for CCR5 binding, inhibiting the recruitment of cancer-promoting cells, thus hindering tumor growth and metastasis.^403–405^ Besides, preclinical results demonstrate that maraviroc could enhance the efficacy of other antitumor agents such as temozolomide and ICB.^406^ Pericyte-derived CCL5 activates CCR5 in glioblastoma cells, triggering DNA-PKcs-mediated DNA damage repair when exposed to temozolomide. Hereto, blocking this CCL5-CCR5 interaction with maraviroc significantly reduces DDR promoted by pericytes and enhances TMZ efficacy in GBM-2 xenografts.^406^ In the phase I trial PICCASSO, the safety and potential antitumor effects of the combination of pembrolizumab and maraviroc were evaluated in patients with refractory mismatch repair proficient colorectal cancer.^407^ Although pembrolizumab combined with maraviroc treatment exhibited a favorable toxicity profile, the ORR was low at 5.3%, and the median PFS was only 2.10 months, with a median OS of 9.83 months.^407^ This early-phase clinical trial suggests the need for further research to enhance therapeutic strategies for this challenging patient population.

**Table 12:** Clinical trials involving CCR5 inhibitors for cancer therapy

Classification	Products	NCT number	Cancer types	Combination partners	Phase	Status
CCR5 antagonist	Maraviroc	NCT04721301	Colorectal and Pancreatic Cancer	Nivolumab and Ipilimumab	I	Completed
		NCT01736813	Colorectal Cancer	Monotherapy	I	Completed
		NCT01785810	Hematologic Malignancy	Monotherapy	II	Completed
		NCT03274804	Colorectal Cancer	Pembrolizumab	I	Completed
		NCT01276236	HIV-related Kaposi’s Sarcoma	Monotherapy	II	Completed
	Vicriviroc	NCT03631407	Colorectal Cancer	Pembrolizumab	II	Completed
Anti-CCR5 mAb	Leronlimab	NCT05730673	CCR5+ Colorectal Cancer	Regorafenib	II	Withdrawn
		NCT04504942	CCR5+ Solid Tumors	Monotherapy	II	Unknown status
		NCT04313075	TNBC	Monotherapy	CU	No longer available
		NCT03838367	TNBC	Monotherapy	I/II	Unknown status

Note: *TNBC* triple negative breast cancer, *CU* compassionate use, *mAb* monoclonal antibody. Clinical trials involving BMS-813160 (CCR2/5 dual antagonist) are present in Table 10

### CXCL8-CXCR1/2 axis blockade

CXCL8, known as IL-8, is produced by a variety of cells including macrophages, epithelial cells, and endothelial cells.^408^ This chemokine, through its cleaved active forms, interacts with its receptors, CXCR1 and CXCR2, to mediate various intracellular signaling pathways such as PI3K-Akt, MAPK, and PLC, influencing cell survival, migration, and angiogenesis.^409–411^ The CXCL8-CXCR1/2 axis plays a pivotal role in cancer by promoting tumor growth, metastasis, and angiogenesis, largely by affecting the TME.^412^ This includes recruiting N2 tumor-associated neutrophils (TANs) and TAMs, influencing the infiltration and function of MDSCs, and promoting the recruitment and proliferation of cancer stem cells, contributing to tumor maintenance, metastasis, and resistance to therapies.^413^ Given its comprehensive role in tumor progression and immune evasion, the CXCL8-CXCR1/2 signaling axis emerges as a promising target for cancer therapy. This is evidenced by the potential benefits of combining anti-CXCL8 antibodies or CXCR1/2 antagonists with conventional anticancer therapies in preclinical models and ongoing clinical trials.^414^

Given the upregulation of CXCL8 and its receptors in various cancers, targeting this axis represents a promising therapeutic strategy to counteract immune suppression within the TME. Small molecule inhibitors and monoclonal antibodies against CXCL8-CXCR1/2 axis such as SB225002, reparixin, navarixin, AZD5069, SX-682, ABX-IL8, and HuMax-IL8 have shown potential in inhibiting tumor progression and enhancing cancer therapy by impairing the recruitment of immunosuppressive cells and angiogenesis (Table 13).^415,416^ For instance, reparixin, targeting CXCR1/2, has inhibited polymorphonuclear cell recruitment and demonstrated a 100-fold higher activity on CXCR1 than CXCR2, highlighting its specificity and potential therapeutic benefit.^417^ In gastric cancer, CXCL8 disrupts CD8^+^ T cell functions by promoting PD-L1 expression on macrophages, while reparixin reduces PD-L1^+^ macrophages and boosts antitumor immunity.^418^ Besides, in the phase I study of anti-CXCL8 antibody HuMax-IL8, while no objective tumor responses were noted, most patients (73%) experienced stable disease, with some maintaining treatment for up to 54 weeks.^419^ Additionally, treatment with HuMax-IL8 led to a significant reduction in serum CXCL8 levels.^419^ These findings underscore the potential of CXCL8 blockade as a strategy to enhance outcomes in cancer therapy, particularly in combination with other immunotherapies. Notably, inspired by the synergistic antitumor activity of CXCL8-CXCR1/2 and ICB in murine tumor models, clinical trials exploring combinations of these inhibitors with PD-1/PD-L1 blockade are underway.^420^ These combination strategies offer new avenues to enhance the efficacy of existing and emerging treatments.

**Table 13:** CXCL8-CXCR1/2 axis blockade for cancer therapy

Classification	Products	NCT number	Cancer types	Combination partners	Phase	Status
Anti-CXCL8 mAb	HuMax-IL8	NCT02536469	Solid Tumor	Monotherapy	I	Completed
		NCT03689699	Prostate Cancer	Nivolumab and Degarelix	I/II	Active, not recruiting
		NCT04848116	HNSCC	Nivolumab	II	Recruiting
		NCT02451982	Pancreatic Cancer	Nivolumab	II	Recruiting
CXCR1/2i	SX-682	NCT05604560	Pancreatic Cancer	Tislelizumab	II	Recruiting
		NCT06228053	mCRPC	Enzalutamide	II	Not yet recruiting
		NCT04574583	Solid Tumors	M7824, MVA-BN-CV301, and FPV-CV301	I/II	Active, not recruiting
		NCT06149481	Colorectal Cancer	Retifanlimab, TriAdeno Vaccine, and N-803	I/II	Not yet recruiting
		NCT04599140	Colorectal Cancer	Nivolumab	I/II	Recruiting
		NCT05570825	NSCLC	Pembrolizumab	II	Recruiting
		NCT04477343	Pancreatic Cancer	Nivolumab	I	Recruiting
		NCT03161431	Melanoma	Pembrolizumab	I	Recruiting
	Ladarixin	NCT05815186	NSCLC With KRAS G12C Mutation	Sotorasib	II	Withdrawn
		NCT05815173	NSCLC With KRAS G12C Mutation	Sotorasib	I	Recruiting
Selective CXCR1i	Reparixin	NCT01861054	Breast Cancer	Monotherapy	II	Terminated
		NCT02001974	Breast Cancer	Paclitaxel	I	Completed
		NCT02370238	Breast Cancer	Paclitaxel	II	Completed
		NCT05212701	Breast Cancer	Monotherapy	II	Withdrawn
Selective CXCR2i	AZD5069	NCT03177187	mCRPC	Enzalutamide	I/II	Terminated
		NCT02499328	Solid Tumors	MEDI4736 and Tremelimumab	I/II	Active, not recruiting
		NCT02583477	Pancreatic Cancer	MEDI4736	I/II	Completed
	Navarixin	NCT03473925	Solid Tumors	Pembrolizumab	II	Completed

Note: *HNSCC* head and neck squamous cell carcinoma, *mCRPC* metastatic castration-resistant prostate cancer, *NSCLC* non-small cell lung cancer

### CXCL12-CXCR4 axis

#### CXCL12-CXCR4 axis promoting tumor growth, metastasis and immune evasion

The CXCL12-CXCR4 axis is pivotal in cancer biology, orchestrating a wide range of processes from tumor growth to metastasis.^421^ CXCL12, also known as stromal cell-derived factor-1 (SDF-1), is a key chemokine that regulates leukocyte trafficking, stem cell homing, and tissue regeneration.^422^ Its interaction with CXCR4, a G-protein coupled receptor expressed on various cell types including cancer cells, activates downstream signaling pathways like Ras, PI3K, and PLC, leading to enhanced cell survival, proliferation, and chemotaxis.^423^ This signaling also involves the activation of JAK-STAT, Wnt-β-catenin, and other pathways, contributing to tumor progression and metastasis.^424,425^ Notably, the CXCL12-CXCR4 axis is pivotal in the intricate regulation of TME, driving the recruitment and infiltration of immunosuppressive cells such as Treg, TAM, and MDSC. These cells contribute to the creation of an immunosuppressive milieu.^426–428^ For instance, the CXCL12-CXCR4 mediated recruitment of TAMs has been linked to increased tumor progression and angiogenesis, while the interaction of CXCR4 with CXCL12 attracts Treg cells, further enhancing the immunosuppressive microenvironment.^429–431^ Targeted inhibition of this signaling pathway, such as the use of the CXCR4 antagonist AMD3100, has shown potential in disrupting these processes, suggesting that modulation of the CXCL12-CXCR4 axis could be a strategic approach to counteract tumor growth, metastasis, and immune evasion mechanisms in cancer therapy.^432,433^

#### CXCL12-CXCR4 inhibitors for cancer therapy

CXCR4 antagonists, initially developed for HIV treatment, have shown promise in the treatment of hematological and solid tumors (Table 14). These inhibitors are categorized into non-peptide antagonists like AMD3100 (Plerixafor), peptide antagonists such as LY2510924, and antibodies like ulocuplumab. AMD3100, the first FDA-approved CXCR4 small-molecule inhibitor, is widely used for stem cell mobilization and harvesting, which has evolved from an immunomodulator to a promising anticancer agent.^434^ Its utility extends beyond monotherapy, showing significant synergies when combined with other anticancer agents, thereby amplifying therapeutic efficacy.^435^ For example, in pancreatic cancer, Feig et al. identified CXCL12 as a critical factor in immunosuppression, produced mainly by FAP^+^ CAFs and preventing T-cell infiltration into tumor regions. Treatment with AMD3100 in combination with anti-PD-L1 led to a significant reduction in tumor growth.^436^ Moreover, in a mouse model of human prostate carcinoma, combining docetaxel with AMD3100 showed a superior antitumor effect compared to docetaxel alone, suggesting that CXCR4 inhibition can effectively chemo-sensitize prostate cancer cells. Further analysis of human prostate cancer samples revealed that cells from bone metastatic lesions exhibited higher levels of CXCR4 than those in primary tumors and lymph node metastases, highlighting the potential of CXCR4 inhibitors as chemo-sensitizing agents.^437^ Furthermore, in vivo models of human TNBC xenografts, AMD3100 treatment notably increased the radiosensitivity of TNBC cells by upregulating Bax, decreasing Bcl-2 levels, inducing prolonged G2-M phase arrest, and elevating apoptosis.^438^ In a phase I/II clinical trial aimed at evaluating the safety and effectiveness of Macrophage Exclusion after Radiation Therapy (MERT) through the administration of AMD3100 in newly diagnosed glioblastoma patients, AMD3100 demonstrated a favorable safety profile with no severe toxicities reported. The median OS was 21.3 months, with the PFS of 14.5 months, suggesting that AMD3100 combined with standard chemo-irradiation could potentially enhance local tumor control in glioblastoma patients.^439^

**Table 14:** CXCL12-CXCR4 axis blockade for cancer therapy

Target	Products	NCT number	Cancer types	Combination partners	Phase	Status
CXCL12	Olaptesed	NCT03168139	Colorectal and Pancreatic Cancer	Pembrolizumab	I/II	Completed
		NCT04901741	Pancreatic Cancer	Pembrolizumab and Chemotherapy	II	Not yet recruiting
		NCT04121455	Glioblastoma	Radiotherapy and Bevacizumab/Pembrolizumab	I/II	Active, not recruiting
		NCT01486797	Chronic Lymphocytic Leukemia	Bendamustine and Rituximab	II	Completed
CXCR4	Plerixafor	NCT04177810	Pancreatic Cancer	Cemiplimab	II	Completed
		NCT00914849	Hematologic Neoplasms	Monotherapy	II	Completed
		NCT04058145	Head and Neck Cancer	Pembrolizumab	II	Withdrawn
		NCT02179970	Pancreatic, Ovarian and Colorectal Cancers	Monotherapy	I	Completed
		NCT01753453	MM	G-CSF	II	Completed
		NCT03277209	Pancreatic Cancer	Monotherapy	I	Terminated
		NCT01288573	Pediatric Cancer	Monotherapy	I/II	Completed
		NCT00241358	Hematological Malignancies	Monotherapy	I/II	Completed
		NCT01225419	Pediatric Cancer	Monotherapy	II	Completed
	MSX-122	NCT00591682	Solid Tumors	Monotherapy	I	Suspended
	BL-8040	NCT02907099	Pancreatic Cancer	Pembrolizumab	II	Completed
		NCT02826486	Pancreatic Cancer	Pembrolizumab and Onivyde	II	Completed
		NCT02639559	Hematological Malignancies	Monotherapy	II	Completed
		NCT04543071	Pancreatic Cancer	Cemiplimab, Gemcitabine, and Nab-Paclitaxel	II	Recruiting
		NCT03246529	MM	G-CSF	III	Active, not recruiting
		NCT03281369	Gastric or Gastroesophageal Junction or Esophageal Cancer	Atezolizumab	I/II	Active, not recruiting
		NCT03193190	Pancreatic Cancer	Atezolizumab	I/II	Active, not recruiting
		NCT01838395	Acute Myeloid Leukemia	Ara-C	II	Completed
		NCT03154827	Acute Myeloid Leukemia	Atezolizumab	I/II	Terminated
		NCT02763384	T-Acute Lymphoblastic Leukemia	Nelarabine	II	Terminated
		NCT02115672	Chronic Myeloid Leukemia	Imatinib	I/II	Withdrawn
	LY2510924	NCT02737072	Solid Tumor	Durvalumab	I	Terminated
		NCT01439568	Extensive Stage Small Cell Lung Carcinoma	Carboplatin and Etoposide	II	Completed
		NCT01391130	ccRCC	Sunitinib	II	Terminated
		NCT02652871	Leukemia	Idarubicin and Cytarabine	I	Completed

Note: *ccRCC* clear cell renal cell carcinoma, *SCLC* small cell lung carcinoma, *MM* multiple myeloma

Additionally, in a phase II trial aimed at enhancing the efficacy of PD-1 inhibitors in pancreatic ductal adenocarcinoma, combining the CXCR4 antagonist BL-8040 (motixafortide) with pembrolizumab and chemotherapy showed promise.^440^ In the first cohort of 37 chemotherapy-resistant patients treated with BL-8040 and pembrolizumab, the DCR of 34.5% was observed, with one individual showing partial response and several others achieving stable disease, leading to the median OS of 3.3 months that extended to 7.5 months for those treated as a second-line option. This treatment also enhanced CD8^+^ effector T cell infiltration, reduced MDSCs, and lowered circulating Tregs. The second cohort, involving 22 patients receiving the triple combination, reported an ORR of 32%, a DCR of 77%, and a median response duration of 7.8 months. These findings indicate that the dual blockade of CXCR4 and PD-1, alongside chemotherapy, could significantly improve outcomes for PDAC patients.^440^ Besides, in a phase IIa clinical trial, the safety and effectiveness of combining BL-8040 with high-dose cytarabine (HiDAC) were assessed in patients with relapsed and refractory acute myelogenous leukemia (AML).^441^ The study explored six escalating doses of BL-8040, ultimately selecting 1.5 mg/kg for an extended evaluation based on safety and tolerability across all levels. Notably, clinical responses were primarily seen at doses of BL-8040 ≥ 1.0 mg/kg, with the composite response rate of 29% across all participants and 39% in those receiving the 1.5 mg/kg dose. The median OS reached 8.4 months across the cohort, extending to 10.8 months for those in the 1.5 mg/kg group and peaking at 21.8 months among responders at this dose.^441^ Initial BL-8040 monotherapy notably mobilized leukemia blasts into the bloodstream, especially in responders, and reduced bone marrow blast counts. These findings highlight the potential of CXCR4 inhibition with BL-8040 as a promising approach for AML treatment, warranting further clinical exploration.^441^

Also, peptide CXCR4 antagonists have similarly blocked CXCR4 in diverse cancer types, showing potential in enhancing immune function and reducing tumor proliferation. In a phase I clinical trial, the safety and efficacy of the peptide antagonist LY2510924 were evaluated in patients with advanced cancers.^442^ Although the best outcome observed was stable disease in 20% of patients, LY2510924 notably increased CD34^+^ cell counts in a dose-dependent manner, achieving up to an 18-fold rise at doses as low as 2.5 mg/day. The findings support LY2510924’s potential for stem cell mobilization with a manageable safety profile, justifying further exploration in phase II trials.^442^ Besides, the combination of peptide CXCR4 antagonist balixafortide and eribulin (chemotherapy agent) demonstrated a safety profile consistent with their monotherapy counterparts and showed promising efficacy in heavily pretreated metastatic breast cancer patients.^443^ Among the 54 evaluable patients, 16 (30%) showed partial responses to the treatment.^443^ Moreover, CXCR4 monoclonal antibodies, including ulocuplumab, have been explored primarily in hematological malignancies, showing the ability to potentiate the effects of other treatments.^444^ Additionally, targeting CXCL12 directly with agents like NOX-A12 impedes the CXCL12-driven movement of CLL cells and renders CLL cells more vulnerable to the chemotherapeutic agents bendamustine and fludarabine in BMSC cocultures.^445^ Overall, the development of CXCL12-CXCR4 axis inhibitors represents a significant advancement in cancer therapy, with ongoing research required to fully understand their potential and integrate them into clinical practice effectively.

### Overexpressing antitumor chemokines or chemokine receptors

Apart from blocking protumor chemokines, overexpressing antitumor chemokines is also a feasible approach to enhancing antitumor immune responses and overcoming the protective mechanisms that tumors use to evade the immune system (Table 15).^344^ One strategy involves increasing the concentration of antitumorigenic chemokines within the TME, either directly or through combination therapies. For instance, chemokines can be synergistically paired with oncolytic viruses (OVs) to boost the recruitment of endogenous effector cells to the tumor site, thereby amplifying the anticancer effects of concurrent therapies.^446^ Preclinical studies have demonstrated the effectiveness of OVs engineered to express chemokines such as CXCL9 or CXCL11, leading to increased infiltration of T and NK cells into tumors, reduced tumor growth, and prolonged survival.^447,448^ Additionally, the development of OVs like NG-641, designed to express a combination of CXCL9, CXCL10, and IFN-α, aims to further enhance the recruitment of immune cells, with clinical trials currently investigating its efficacy in patients with advanced solid tumors.^449,450^

**Table 15:** Overexpressing antitumor chemokines or chemokine receptors for cancer therapy

Products	NCT number	Cancer types	Combination partners	Phase	Status
NG-641 (Oncolytic adenoviral producing a FAP-targeting bispecific T cell activator and cytokines CXCL9, CXCL10, and IFN-α2)	NCT05043714	Epithelial Tumor	Nivolumab	I	Recruiting
	NCT04053283	Epithelial Tumor	Monotherapy	I	Recruiting
	NCT04830592	HNSCC	Pembrolizumab	I	Recruiting
CD19-7×19 CAR-T (Anti-CD19 CAR-T Expressing IL-7 and CCL19)	NCT04833504	B Cell Lymphoma	Monotherapy	I	Completed
	NCT05659628	DLBCL	Tislelizumab	I	Recruiting
	NCT04381741	DLBCL	Anti-PD1 mAb	I	Enrolling by invitation
CCL21-Gene Modified Dendritic Cell Vaccine	NCT03546361	NSCLC	Pembrolizumab	I	Recruiting
	NCT01574222	NSCLC	Monotherapy	I	Terminated
	NCT00601094	Lung Cancer	Monotherapy	I	Completed
	NCT00798629	Melanoma	Monotherapy	I	Completed
CCL21 protein	NCT01433172	Lung Cancer	GM.CD40L Vaccine	I/II	Completed
CXCR4 Modified Anti-CD30 CAR-T	NCT03602157	CD30^+^ Lymphoma	Monotherapy	I	Recruiting
CXCR4 Modified Anti-BCMA CAR-T	NCT04727008	Multiple Myeloma	Monotherapy	I	Recruiting
CXCR4 Modified Anti-CD19 CAR-T	NCT04684472	CD19^+^ B-cell Malignancies	Monotherapy	I	Recruiting
CXCR5 modified Anti-EGFR CAR-T	NCT04153799	NSCLC	Monotherapy	I	Unknown status
CXCR5 modified Anti-EGFR CAR-T	NCT05060796	NSCLC	Monotherapy	I	Recruiting

Note: *HNSCC* head and neck squamous cell carcinoma, *DLBCL* diffuse large B-cell lymphoma, *NSCLC* non-small cell lung cancer, *CAR-T* chimeric antigen receptor T-cell

Another promising avenue involves the administration of fusion proteins that link chemokines with antibodies or other targeting molecules, directing these immune-modulating agents specifically to tumor cells or the tumor stroma. This approach has led to the development of chemokine-antibody fusion proteins that target specific tumor antigens, such as CXCL10-EGFRvIII for glioma or an anti-human endoglin scFv fused to CXCL10 for hepatocellular carcinoma, showing promising results in enhancing intratumoral effector cell recruitment and improving antitumor activity in preclinical models.^451^ Similarly, the use of chemokines as adjuvants in cancer vaccines has been explored, with chemokines like CCL21 being employed to boost the recruitment and activation of DCs and T cells, enhancing the efficacy of cancer vaccines in preclinical models, and leading to clinical trials assessing their utility in various cancer types.^452–454^ Moreover, the direct genetic modification of therapeutic cells to overexpress chemokines or chemokine receptors has emerged as a novel strategy to improve cellular therapies for cancer. By engineering CAR-T cells to co-express chemokines such as CCL19 or chemokine receptors like CXCR6, these modified cells can more effectively home to tumor sites and interact with endogenous immune cells, leading to enhanced antitumor responses.^455–457^ Notably, synthetic biology provides an innovative approach for the targeted delivery of chemokines directly into the TME. This novel strategy overcomes immune cell exclusion by deploying engineered bacteria that intratumorally release specific chemokines, like an activating mutant of human CXCL16 (hCXCL16^K42A^), to attract adaptive immune cells to tumors.^458^ This hCXCL16^K42A^ expressing bacteria (eSLC-hCXCL16^K42A^) showed significant therapeutic potential in multiple tumor models, primarily by recruiting CD8^+^ T cells.^458^ Additionally, the eSLC-hCXCL16^K42A^ strain synergized with CCL20-expressing bacteria (eSLC-CCL20) to boost antitumor immunity, by simultaneously improving the recruitment of cDC1 and CD8^+^ T cells, eventually overcoming immunotherapy resistance in immune-excluded tumors.^458^

In a phase I clinical trial of NCT03198546, the safety and efficacy of CAR-T cells secreting IL-7 and CCL19 (7×19) were evaluated in patients with advanced hepatocellular carcinoma, pancreatic carcinoma, and ovarian carcinoma expressing glypican-3 (GPC3) or mesothelin (MSLN).^459^ Notably, one hepatocellular carcinoma patient treated with anti-GPC3-7×19 CAR-T cells achieved complete tumor remission 30 days after intratumoral injection, and a pancreatic carcinoma patient treated with anti-MSLN-7×19 CAR-T cells experienced almost complete tumor remission 240 days after intravenous infusion.^459^ These findings suggest that incorporating IL-7 and CCL19 into CAR-T cell therapy significantly boosts its efficacy against solid tumors, marking a significant advancement in the field. Currently, more clinical trials are underway to evaluate the efficacy of these modified CAR-T cells in treating a range of hematological and solid tumors, demonstrating the potential of chemokines to significantly improve the therapeutic landscape of cancer treatment through various innovative approaches.

## Growth factor blockade

The growth factor is a type of cytokine that specifically plays a role in the regulation of cell growth, proliferation, and differentiation. Growth factors like TGF-β, VEGF, and EGF play pivotal roles in cancer progression through the promotion of angiogenesis, tumorigenesis, and metastasis.^460–463^ The investigation into these growth factors has been instrumental in developing targeted therapies, offering a more personalized treatment approach for cancer patients. Inhibitors targeting TGF-β, VEGF, and EGFR have shown significant promise in clinical settings.

### TGF-β inhibition

#### TGF-β signaling and its dual role in cancer

TGF-β is a key cytokine in the TGF-β superfamily, encompassing TGF-βs, Activins, Nodals, BMPs, and GDFs, pivotal in embryogenesis and adult physiological homeostasis.^464^ It exists as three mammalian isoforms (TGF-βI-III).^465^ For clarity, discussions around TGF-β typically refer to TGF-βI unless specified otherwise. TGF-β is synthesized and secreted into the extracellular matrix (ECM) predominantly in a latent complex form.^466,467^ The molecule undergoes a sophisticated activation process, initiated by cleavage via the convertase enzyme furin within the Golgi apparatus, which separates the latency-associated peptide (LAP) from the mature TGF-β cytokine, albeit maintaining a non-covalent association that keeps TGF-β inactive until further activation cues are met.^468^ Then, with the assistance of mechanical forces and αβ integrins, inactive TGF-β is activated and binds to the receptor complex, initiating the regulation of gene transcription via SMAD and non-SMAD pathways.^469,470^

**Fig. 7:**
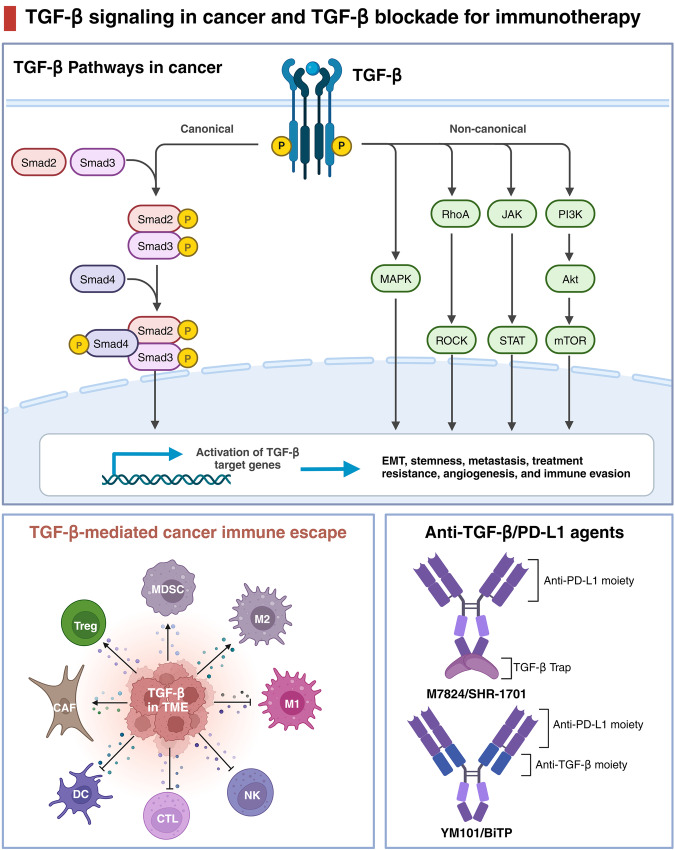
TGF-β signaling in cancer and TGF-β blockade for immunotherapy. The top panel illustrates the TGF-β signaling pathway in cancer cells, including the canonical Smad-dependent pathway and the non-canonical pathways involving various intracellular mediators such as MAPK, PI3K/Akt, and mTOR, leading to cellular processes like EMT, stemness, metastasis, treatment resistance, angiogenesis, and immune evasion. The bottom left panel depicts the role of TGF-β in the tumor microenvironment (TME), highlighting its immunosuppressive effects that facilitate cancer immune escape by interacting with various immune cells such as Treg, MDSC, M1/M2 macrophages, DC, NK, and CTL. The bottom right panel presents a schematic representation of innovative anti-TGF-β/PD-L1 therapeutic agents, demonstrating dual blockade strategies, as exemplified by M7824/SHR-1701, which combines a TGF-β trap with an anti-PD-L1 moiety, and YM101/BITP, which features both anti-TGF-β and anti-PD-L1 moieties for enhanced immunotherapy efficacy. Adapted from “Canonical and Non-canonical TGF-β Pathways in EMT”, by BioRender.com (2024). Retrieved from https://app.biorender.com/biorender-templates

Specifically, TGF-β signaling initiates when TGF-β ligands bind to type II receptors (TGFβRII), leading to the activation and phosphorylation of type I receptors (TGFβRI).^471^ This triggers the phosphorylation of SMAD2 and SMAD3, which then form trimeric complexes with SMAD4.^472^ These complexes enter the nucleus to regulate genes such as TWIST1, SNAI1, and SNAI2, impacting cellular functions like proliferation and differentiation.^473,474^ Beyond this canonical pathway, TGF-β also activates non-SMAD pathways, including the PI3K-AKT, MAPK, and RHO signaling (Fig. 7).^475,476^ The dysregulation of TGF-β signaling is implicated in a myriad of pathological conditions, including metabolic dysfunctions, excessive ECM deposition, immune dysfunction, fibrosis, and various cancers.^477^ In cancer, TGF-β exhibits dual roles, initially suppressing tumor formation by halting the cell cycle, but in advanced stages, it aids tumor growth by promoting EMT, increasing metastasis, chemoresistance, angiogenesis, and immune evasion.^478^ This switch from a tumor suppressor to a promoter is a key feature in the progression of advanced cancers, underscoring the complex nature of TGF-β in oncogenesis.^479^

#### TGF-β inhibition for improved cancer immunotherapy response

The targeting of TGF-β signaling has become a focal point in cancer therapy, given its role in fostering immune evasion and resistance to immunotherapies by altering the TME.^480^ Multiple TGF-β-targeted therapies, including monoclonal antibodies, ligand traps, receptor kinase inhibitors, antisense oligonucleotides, and vaccines, are currently under clinical investigation (Table 16).^5,481,482^ Fresolimumab (also known as GC-1008), a monoclonal antibody against TGF-β, demonstrated promising antitumor activities in renal cell carcinoma and melanoma (NCT00356460).^483^ Besides, the safety, efficacy, and immune responses of fresolimumab combined with radiotherapy were investigated in patients with metastatic breast cancer.^484^ Participants were assigned to receive either 1 mg/kg or 10 mg/kg fresolimumab every three weeks for five cycles, alongside focal radiotherapy targeting a metastatic site. Patients administered the 10 mg/kg dose of fresolimumab exhibited a significantly longer median OS compared to those on the 1 mg/kg dose, with the HR of 2.73 (95% CI, 1.02–7.30; *P* = 0.039).^484^ Additionally, the higher dose was associated with enhanced peripheral blood mononuclear cell counts and a notable increase in the CD8^+^ central memory T cell pool.^484^ The results suggest that TGF-β blockade combined with radiotherapy is a viable and safe strategy, with the higher fresolimumab dose prompting a more favorable systemic immune response and improved survival outcomes.^484^

**Table 16:** Agents targeting TGF-β in preclinical or clinical studies

Target	Molecular type	Agent	Company
TGFβRII	mAb	LY3022859	Eli Lilly
TGFβRI/II	Receptor kinase inhibitor	LY2109761	Eli Lilly
TGFβRI	Receptor kinase inhibitor	Vactosertib	MedPacto
		Galunisertib	Eli Lilly
		LY3200882	Eli Lilly
		LY573636	Eli Lilly
		SB-431542	GlaxoSmithKline
		SB-505124	GlaxoSmithKline
		IN-1130	In2Gen
TGF-β and PD-L1	BsAb	BiTP/YM101	YZY Biopharma
TGFβRII and PD-L1	Bifunctional fusion protein	TQB2858	Chia-Tai Tianqing
		M7824	Merck KGaA
		PM8001	Pumis Biotechnology
		SHR-1701	Hengrui
TGFβRII and PD-1	Bifunctional fusion protein	JS201	Junshi
TGF-β and VEGF	BsAb	Y332	YZY Biopharma
TGF-β	mAb	Fresolimumab	Genzyme
		SRK181	Scholar Rock
		1D11	Genzyme
		2G7	Genentech
	Trap	AVID200	Forbius
		Luspatercept	Acceleron
	Antisense oligonucleotides	AP 12009	Antisense Pharma
		AP 11014	Antisense Pharma
	Cancer vaccine	Vigil	Gradalis
		Lucanix	NovaRx
Integrin αvβ6	mAb	264RAD	AstraZeneca

Note: *BsAb* bispecific antibody, *mAb* monoclonal antibody

Notably, galunisertib, a TGFβRI inhibitor, when combined with gemcitabine, enhanced OS in pancreatic cancer patients, marking a significant advancement over gemcitabine monotherapy.^485^ In this clinical trial for patients with unresectable pancreatic cancer, the primary endpoint of OS was achieved, with median OS of 8.9 months for the combination group and 7.1 months for the gemcitabine group (HR = 0.79).^485^ Moreover, galunisertib combined with neoadjuvant chemoradiotherapy was effective in patients with locally advanced rectal adenocarcinoma.^486^ In this phase II trial, out of 38 enrolled patients, 25 proceeded to surgery after completing chemoradiotherapy, with 20% achieving pathological complete responses.^486^ Ten patients opted for non-operative management, with 71% showing clinical complete responses after one year. Overall, 32% of patients achieved a complete response. The treatment was generally well-tolerated, with common grade 3 adverse events being diarrhea and hematological toxicity.^486^ However, in a study evaluating the combination of galunisertib and lomustine in patients with glioblastoma, no improvement in OS was observed compared to placebo plus lomustine.^487^ Similarly, the phase Ib study on advanced hepatocellular carcinoma patients combining galunisertib and ramucirumab found the treatment safe but with limited efficacy, leading to the discontinuation of further clinical development of this combination.^488^

The disparate outcomes of clinical trials exploring galunisertib combinations can be attributed to tumor heterogeneity, variations in patient demographics and disease stages, differences in drug dosing and pharmacokinetics, interactions between TGF-β and other cellular pathways, and study design specifics.^489^ These factors highlight the complexity of TGF-β targeted therapies and the necessity for tailored treatment strategies and further mechanistic studies. At present, there are more than ten TGFβRI inhibitors are undergoing clinical evaluation. For instance, despite tolerable toxicity in clinical trials, LY573636 by Eli Lilly showed only modest antitumor effects in NSCLC patients, highlighting the challenge of translating TGF-β receptor kinase inhibitors’ preclinical success into clinical efficacy.^490^

#### Novel bifunctional antibodies simultaneously targeting PD-L1 and TGF-β

Moreover, M7824, a bifunctional fusion protein targeting both PD-L1 and TGF-β pathways, has shown promising antitumor activity in preclinical and early clinical trials, highlighting its potential in reprogramming the TME and reversing immunotherapy resistance (Table 17).^491^ In the phase I trial of M7824 (NCT02517398), 19 heavily pretreated patients with advanced solid tumors were treated with doses up to 20 mg/kg every 2 weeks.^492^ Efficacy signals included one ongoing complete response in cervical cancer, two confirmed partial responses in pancreatic and anal cancers, one near-partial response in cervical cancer, and two instances of prolonged stable disease in pancreatic cancer and carcinoid.^492^ Besides, in expansion cohort of NCT02517398, 80 patients with advanced NSCLC received either 500 mg or 1200 mg doses, achieving an overall response rate of 21.3%.^493^ The 1200 mg dose showed a higher response rate, especially in PD-L1-positive patients, with an ORR of 36.0% and 85.7% in those with PD-L1-high expression.^493^ Treatment-related adverse events were reported in 69% of patients, with 29% experiencing grade 3 or higher events, and 10% discontinued treatment due to adverse events.^493^ These results highlight M7824’s manageable safety profile and its promising early signs of efficacy in advanced solid tumors. Similarly, SHR-1701, another fusion protein combining anti-PD-L1 antibody with a TGF-β trap, has shown promising antitumor effects in various cancers especially gastric cancer and cervical cancer.^494,495^

**Table 17:** Clinical trials of bifunctional fusion protein M7824 targeting PD-L1 and TGF-β

NCT number	Cancer types	Combination partners	Phase	Status
NCT03833661	Biliary Tract Cancer	Monotherapy	II	Completed
NCT03631706	NSCLC	Monotherapy	III	Active, not recruiting
NCT04835896	Gastric Cancer	Paclitaxel	I/II	Not yet recruiting
NCT03840902	NSCLC	Concurrent Chemoradiotherapy	II	Terminated
NCT03554473	SCLC	Topotecan or Temozolomide	I/II	Recruiting
NCT04574583	Solid Tumors	SX-682 and CV301	I/II	Active, not recruiting
NCT04296942	Breast Cancer	BN-Brachyury, Entinostat, and Adotrastuzumab Emtansine	I	Terminated
NCT05145569	Ovarian Cancer	Carboplatin AUC 5 and paclitaxel	I	Not yet recruiting
NCT02699515	Solid Tumors	Monotherapy	I	Completed
NCT04327986	Pancreatic Cancer	M9241 and SBRT	I/II	Terminated
NCT02517398	Solid Tumors	Monotherapy	I	Completed
NCT03524170	Breast Cancer	Radiation Therapy	I	Completed
NCT03427411	HPV Associated Malignancies	Monotherapy	II	Completed
NCT03620201	HER2 Positive Breast Cancer	Monotherapy	I	Active, not recruiting
NCT03436563	Solid Tumors With Microsatellite Instability	Monotherapy	I/II	Active, not recruiting
NCT04489940	TNBC	Monotherapy	II	Terminated
NCT04246489	Cervical Cancer	Monotherapy	II	Completed
NCT03579472	TNBC	Eribulin Mesylate	I	Terminated
NCT04432597	HPV Associated Malignancies	HPV Vaccine PRGN-2009	I/II	Active, not recruiting
NCT04235777	Non-Prostate Genitourinary Malignancies	M9241 and SBRT	I	Recruiting
NCT04066491	Biliary Tract Cancer	Gemcitabine and Cisplati	II/III	Terminated
NCT05445882	Castration Resistant Prostate Cancer	N-803 and BN-Brachyury	II	Not yet recruiting
NCT04417660	Thymic Cancer	Monotherapy	II	Recruiting
NCT04501094	Urothelial Cancer	Monotherapy	II	Terminated
NCT04247282	HNSCC	TriAd Vaccine and N-803	I/II	Completed
NCT03840915	NSCLC	Chemotherapy	I/II	Completed
NCT04287868	HPV Associated Malignancies	PDS0101 and NHS-IL12	I/II	Active, not recruiting
NCT04551950	Cervical Cancer	Cisplatin/Carboplatin, Paclitaxel, and Bevacizumab	I	Completed
NCT04727541	Biliary Tract Cancer	Monotherapy	II	Terminated
NCT04560686	NSCLC	Surgery	II	Terminated
NCT05061823	Lung Cancer	Monotherapy	III	Active, not recruiting
NCT03451773	Pancreatic Cancer	Gemcitabine	I/II	Terminated
NCT03493945	Solid Tumor	BN-Brachyury Vaccine, N-803 and Epacadostat	I/II	Recruiting
NCT04349280	Urothelial Cancer	Monotherapy	I	Active, not recruiting
NCT04633252	Prostate Cancer	ADT, Prednisone, Docetaxel and M9241	I/II	Recruiting
NCT04491955	Small Bowel and Colorectal Cancers	CEA/MUC1 Vaccines, N-803, and NHSIL12	II	Active, not recruiting
NCT03315871	Prostate Cancer	PROSTVAC-V and CV301	II	Active, not recruiting
NCT05012098	Olfactory Neuroblastoma	Monotherapy	II	Active, not recruiting
NCT04220775	HNSCC	SBRT	I/II	Completed
NCT04756505	Breast Cancer	NHS-IL12 and Radiotherapy	I	Withdrawn
NCT04396535	NSCLC	Docetaxel	II	Terminated
NCT04789668	Brain Metastases	Pimasertib	I/II	Completed
NCT04708470	HPV-Associated Malignancies, Small Bowel, and Colon Cancers	NHS-IL12 and Entinostat	I/II	Recruiting
NCT04648826	Pulmonary Metastases	Azacytidine	I/II	Withdrawn
NCT04971187	TKI-Resistant EGFR-Mutant NSCLC	Pemetrexed and Carboplatin/Cisplatin	II	Terminated
NCT05005429	Malignant Pleural Mesothelioma	Monotherapy	II	Recruiting
NCT04303117	Kaposi Sarcoma	NHS-IL12	I/II	Recruiting
NCT04428047	HNSCC	Monotherapy	II	Terminated
NCT04708067	Intrahepatic Cholangiocarcinoma	Hypofractionated Radiotherapy	I	Active, not recruiting

Note: *SBRT* stereotactic body radiation therapy, *ADT* androgen deprivation therapy, *NSCLC* non-small cell lung cancer, *SCLC* small cell lung cancer, *TNBC* triple negative breast cancer, *HNSCC* head and neck squamous cell carcinoma, *TKI* tyrosine kinase inhibitor

In parallel with the fusion protein, YM101, an innovative anti-TGF-β/PD-L1 bispecific antibody developed from the Check-BODY™ technology platform, has demonstrated the capacity to specifically target TGF-β and PD-L1, counteracting their immunosuppressive effects in vitro and showing superior antitumor activity in vivo compared to monotherapies targeting either pathway alone.^496,497^ By promoting an immune-supportive TME, which was characterized by increased infiltration of lymphocytes and dendritic cells, a higher M1/M2 macrophage ratio, and elevated cytokine production in T cells, YM101 effectively enhances the antitumor immune response, offering a promising strategy to overcome resistance and enhance the efficacy of anti-PD-1/PD-L1 therapies.^496^ Besides, the combination of bivalent manganese, a natural STING agonist, with YM101 has demonstrated a synergistic antitumor effect in preclinical studies, effectively transforming immune-excluded and immune-desert tumor models into immune-inflamed ones by activating both innate and adaptive immune responses, enhancing DC maturation, T cell activation, and antigen presentation.^73^ Similarly, MSA-2, another novel STING agonist, when combined with YM101, significantly improved antitumor activity in these resistant tumor models by promoting proinflammatory cytokine and chemokine production, boosting antigen presentation, and increasing tumor-infiltrating lymphocytes, showcasing the potential of these combinations as universal regimens for treating various tumor immune landscapes.^72^

Lastly, antisense oligonucleotides and cancer vaccines offer innovative strategies targeting TGF-β in cancer therapy. AP 12009, an antisense oligodeoxynucleotide developed by Antisense Pharma, targets TGF-β2 and has shown improved OS for high-grade glioma.^498^ Notably, in a prespecified subgroup analysis of patients with anaplastic astrocytoma, the 10 µM trabedersen group demonstrated a significant improvement in the 14-month tumor control rate compared to chemotherapy (*P* = 0.0032).^498^ Additionally, this group showed a trend towards superior 2-year survival (*P* = 0.10), with median OS of 39.1 months for 10 µM trabedersen, 35.2 months for 80 µM trabedersen, and 21.7 months for chemotherapy.^498^ In the realm of cancer vaccines, belagenpumatucel-L, a vaccine by NovaRx composed of NSCLC cells with a TGF-β2 antisense gene, demonstrated improved survival in NSCLC patients in a phase III trial.^499^ The overall trial did not meet its primary endpoint, with median survival at 20.3 months for belagenpumatucel-L versus 17.8 months for placebo.^499^ However, prespecified analyses showed patients randomized within 12 weeks after chemotherapy and those who received prior radiation benefited from belagenpumatucel-L, with median survival extending to 28.4 months compared to 16.0 months for placebo recipients in the radiation subgroup.^499^ In sum, these clinical outcomes underscore the critical role of TGF-β-targeted therapies in the evolving cancer treatment paradigm.

### Blocking pro-angiogenic factors

#### Abnormal angiogenesis in cancer and its role in immune evasion

Angiogenesis, the formation of new blood vessels from pre-existing vasculature, is a crucial process in both physiological conditions, such as wound healing, and pathological conditions, including cancer development and metastasis.^500^ The rapid proliferation of tumor cells increases the demand for oxygen and nutrients, leading to hypoxia and acidosis within the TME.^501^ This condition triggers the secretion of various pro-angiogenic factors like VEGF, MMPs, and basic fibroblast growth factor (bFGF), disrupting the balance between pro-angiogenic and anti-angiogenic factors and activating angiogenic pathways.^502^ However, the continuous overproduction of these factors results in the formation of abnormal, immature blood vessels characterized by a lack of pericyte coverage and increased leakiness, which contributes to elevated vascular permeability and interstitial fluid pressure, further hampering drug delivery and immune cell infiltration into tumors.^503–506^

**Fig. 8:**
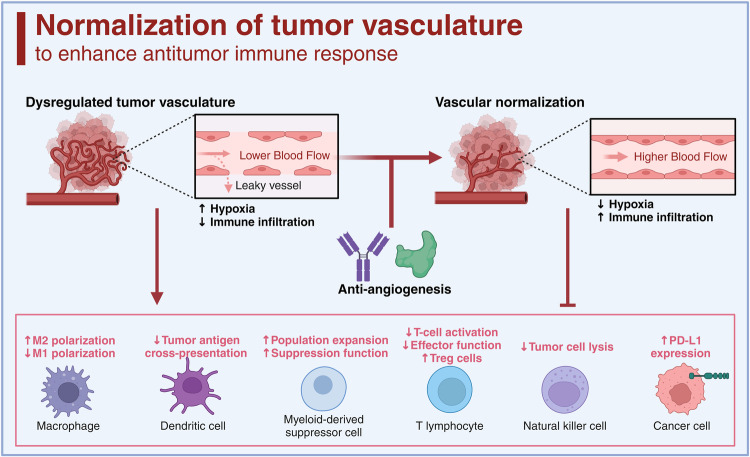
Anti-angiogenesis therapy to improve antitumor immune response. The left side of the figure depicts the consequences of dysregulated tumor vasculature, characterized by lower blood flow, leaky vessels, and resulting hypoxia that subsequently leads to decreased immune infiltration, increased M2 macrophage polarization, reduced M1 polarization, diminished tumor antigen cross-presentation by dendritic cells, expansion and enhanced suppression function of myeloid-derived suppressor cells, along with inhibited T-cell activation, effector function, and increased regulatory T (Treg) cell populations. The introduction of anti-angiogenesis treatment targets these aberrant vessels to shift the balance towards vascular normalization, as shown on the right. This normalization results in improved blood flow, reduced hypoxia, and increased immune infiltration, thereby potentially increasing T-cell activation, enhancing effector function, promoting tumor cell lysis by natural killer cells, and reducing PD-L1 expression on cancer cells, collectively creating an optimized microenvironment for the antitumor immune response. (Created with BioRender.com)

While the primary goal of anti-angiogenic therapy was to deprive tumor cells of their blood supply, standalone treatments have not significantly improved patient outcomes, suggesting a need for combined therapeutic strategies.^507^ The concept of vessel normalization has emerged, proposing a synergistic effect when anti-angiogenic therapies are used in combination with other treatments, such as ICB (Fig. 8).^508^ This approach aims to modulate the tumor vasculature to improve perfusion and oxygenation, reducing hypoxia-induced immunosuppression and enhancing the efficacy of immunotherapies.^509^ Abnormal angiogenesis not only supports tumor growth and metastasis but also plays a pivotal role in immune evasion by hindering the infiltration and function of immune cells within the TME.^501^ The excessive production of angiogenic factors not only promotes the growth of leaky and disorganized blood vessels but also directly contributes to the suppression of antitumor immune responses. VEGF, for instance, can directly inhibit the trafficking, proliferation, and effector functions of CTLs.^510^ Furthermore, VEGF impedes the maturation and antigen-presenting capability of DCs, crippling the activation of T cells and, consequently, dampening the immune response to tumor antigens.^511^ Besides, the hypoxia TME promotes the accumulation of immunosuppressive cells like Tregs, MDSCs, and TAMs that exhibit protumor activities.^512–514^ These immunosuppressive cells further secrete cytokines and growth factors, including more VEGF and TGF-β, reinforcing the cycle of angiogenesis and immunosuppression. Additionally, angiogenic molecules can modulate the expression of immune checkpoint molecules, such as PD-L1 on tumor and immune cells, and adhesion molecules on endothelial cells, which further diminish the effectiveness of CTLs and facilitate tumor immune evasion.^515–518^ This complex interplay between angiogenesis and immune suppression underscores the challenges in treating cancers solely with anti-angiogenic or immunotherapeutic agents and highlights the potential benefits of combining these therapeutic strategies to normalize tumor vasculature, alleviate immunosuppression, and enhance the efficacy of cancer immunotherapy.^519^

#### Anti-angiogenesis agents and their applications in cancer immunotherapy

In preclinical studies, the synergistic effect of anti-angiogenesis therapy combined with ICB has been increasingly recognized as a potent strategy against cancer. Anti-angiogenesis therapy, aimed at normalizing tumor vasculature, not only inhibits tumor growth by disrupting blood supply but also enhances the efficacy of ICB.^520^ This synergy has been observed in various preclinical models, including melanoma, colon, kidney, breast, and lung cancers, where combination therapy significantly prolonged the OS of tumor-bearing mice.^521–524^ Key mechanisms underlying this synergy include the normalization of tumor vessels, which improves T cell infiltration and reprograms the TME from immunosuppressive to immune-supportive phenotype.^525^ This effect is achieved by reducing hypoxia and downregulating immune checkpoint expression on T cells and tumor cells.^510,522,526^ Furthermore, the formation of high endothelial venules (HEV) after combination therapy has been identified as a novel mechanism that promotes lymphocyte homing and infiltration into the tumor.^527^ These findings underscore the complexity and potential of combining ICB with anti-angiogenesis therapy for cancer treatment, highlighting the need for further exploration to fully understand and optimize this therapeutic strategy.

Encouraged by promising preclinical findings, extensive clinical research has been undertaken to explore the combined efficacy of ICB with anti-angiogenesis treatments (Table 18). Clinical trials, such as the phase I study NCT00790010, have begun to unveil the potential of combining ICB like ipilimumab (anti-CTLA-4) with anti-VEGF agents such as bevacizumab in treating metastatic melanoma,^528^ demonstrating significant improvements in prognosis and enhanced immune responses. Administering ipilimumab combined with bevacizumab across four dosing cohorts to forty-six patients, the research observed inflammatory responses, enhanced endothelial activation, and significant immune cell infiltration within tumors.^528^ Additionally, improvements in peripheral blood markers, including increased CCR7^+/−^CD45RO^+^ cells and anti-galectin antibodies, were detected.^528,529^ The combination treatment yielded a DCR of 67.4%, with 8 partial responses and 22 instances of stable disease, culminating in a median survival of 25.1 months. These outcomes underscore the potential of bevacizumab to modify tumor vasculature and immune dynamics in concert with ipilimumab, offering a promising therapeutic strategy that combines angiogenesis inhibition with ICB.^528^

**Table 18:** Representative clinical trials of the combination therapy of anti-angiogenesis and immune checkpoint blockade

Combination strategy	NCT number	Cancer types	Phases	Status
Atezolizumab combined with Bevacizumab	NCT05063552	HNSCC	II/III	Recruiting
	NCT02366143	NSCLC	III	Completed
	NCT04732598	Breast Cancer	III	Active, not recruiting
	NCT03038100	Ovarian, Fallopian Tube, or Primary Peritoneal Cancer	III	Completed
	NCT03353831	Ovarian Cancer	III	Active, not recruiting
	NCT02997228	Colorectal Adenocarcinoma	III	Recruiting
	NCT05665348	HCC	II/III	Not yet recruiting
	NCT03991403	NSCLC	III	Active, not recruiting
	NCT04194203	NSCLC	III	Active, not recruiting
	NCT03434379	HCC	III	Completed
	NCT04487067	HCC	III	Active, not recruiting
	NCT03693573	RCC	III	Withdrawn
	NCT05904886	HCC	III	Recruiting
	NCT02420821	RCC	III	Completed
	NCT04803994	HCC	III	Recruiting
	NCT04732286	HCC	III	Active, not recruiting
	NCT04712643	HCC	III	Active, not recruiting
	NCT04102098	HCC	III	Active, not recruiting
	NCT03556839	Cervical Cancer	III	Active, not recruiting
Axitinib Combined with Avelumab	NCT03013946	RCC	III	Recruiting
	NCT03472560	NSCLC and Urothelial Cancer	II	Terminated
	NCT02912572	Endometrial Cancer	II	Active, not recruiting
	NCT04258956	Gastrointestinal Stromal Tumors	II	Unknown status
	NCT02684006	RCC	III	Active, not recruiting
	NCT03386929	NSCLC	I/II	Terminated
	NCT04562441	NPC	II	Active, not recruiting
	NCT04698213	RCC	II	Recruiting
	NCT05327686	RCC	II	Recruiting
	NCT03990571	Adenoid Cystic Carcinoma	II	Completed
	NCT03341845	RCC	II	Recruiting
	NCT03291314	Recurrent Glioblastoma	II	Completed
	NCT05176288	ccRCC	II	Withdrawn
	NCT05249569	HCC	II	Terminated
Pembrolizumab Combined With Lenvatinib	NCT04676412	NSCLC	III	Active, not recruiting
	NCT03829332	NSCLC	III	Active, not recruiting
	NCT04716933	NSCLC	III	Active, not recruiting
	NCT03829319	NSCLC	III	Active, not recruiting
	NCT03976375	NSCLC	III	Active, not recruiting
	NCT03898180	Urothelial Carcinoma	III	Active, not recruiting
	NCT03517449	Endometrial Neoplasms	III	Active, not recruiting
	NCT04776148	Colorectal Neoplasms	III	Active, not recruiting
	NCT04949256	Esophageal Carcinoma	III	Recruiting
	NCT05077215	Endometrial Cancer	III	Not yet recruiting
	NCT04865289	Endometrial Cancer	III	Active, not recruiting
	NCT03884101	Endometrial Cancer	III	Active, not recruiting
	NCT03486873	Solid and Hematologic Malignancies	III	Recruiting
	NCT04889118	Melanoma	III	Active, not recruiting
	NCT03820986	Melanoma	III	Active, not recruiting
	NCT05899049	RCC	III	Recruiting
	NCT04736706	RCC	III	Recruiting
	NCT04662710	Gastroesophageal Adenocarcinoma	III	Active, not recruiting
	NCT05523323	HNSCC	III	Active, not recruiting
	NCT04199104	HNSCC	III	Active, not recruiting
	NCT03713593	HCC	III	Active, not recruiting
	NCT04246177	HCC	III	Active, not recruiting
	NCT02811861	RCC	III	Active, not recruiting
SHR-1210 Combined with Apatinib	NCT03813784	Gastric Cancer	III	Unknown status
	NCT04335006	TNBC	III	Terminated
	NCT04342910	Gastric Cancer	III	Unknown status
	NCT05049681	Esophageal Cancer	III	Unknown status
Nivolumab Combined with Sitravatinib	NCT03906071	NSCLC	III	Active, not recruiting
	NCT03015740	Kidney Cancer	I/II	Completed
	NCT04904302	ccRCC	II	Active, not recruiting
	NCT02954991	NSCLC	II	Completed
	NCT04887870	Solid Malignancies	II/III	Enrolling by invitation
	NCT03606174	Urothelial Carcinoma	II	Terminated
	NCT03680521	ccRCC	II	Completed

Note: *HNSCC* head and neck squamous cell carcinoma, *NSCLC* non-small-cell lung cancer, *HCC* hepatocellular carcinoma, *RCC* renal cell carcinoma, *ccRCC* clear cell renal cell cancer, *NPC* nasopharyngeal carcinoma, *TNBC* triple-negative breast cancer

Besides combination therapies with anti-CTLA-4, several clinical trials have investigated the combination of anti-PD-L1 with anti-VEGF therapies across different cancer types, showing promising results. The phase II/III clinical trial ORIENT-32 aimed to assess the efficacy and safety of combining sintilimab, an anti-PD-L1 antibody, with IBI305, a bevacizumab biosimilar, versus sorafenib for the first-line treatment of unresectable HBV-associated hepatocellular carcinoma.^530^ In the phase II part, 24 patients received at least one dose of the study drugs, achieving the ORR of 25%, with grade 3 or worse treatment-related adverse events in 29% of patients.^530^ This led to the commencement of the phase III part, where the sintilimab-bevacizumab biosimilar group showed the median PFS of 4.6 months compared to 2.8 months in the sorafenib group (HR: 0.56, *P* < 0.0001).^530^ The first interim analysis for OS indicated a significant advantage for the combination therapy, with a median survival not yet reached, versus 10.4 months for sorafenib (HR: 0.57, *P* < 0.0001).^530^ Adverse events were manageable, with hypertension (14% vs 6%) and palmar-plantar erythrodysesthesia syndrome (0% vs 12%) being the most common grade 3–4 events in the sintilimab-bevacizumab biosimilar and sorafenib groups, respectively^530^. These results suggest that sintilimab plus IBI305 could offer a substantial survival benefit with a tolerable safety profile for patients with HBV-associated hepatocellular carcinoma, presenting a promising first-line treatment alternative.^530^

Also, in the phase II trial NCT02873962, the efficacy of combining nivolumab and bevacizumab was evaluated in 38 women with relapsed epithelial ovarian cancer.^531^ The primary endpoint ORR was 28.9% overall, with a higher ORR observed in platinum-sensitive patients (40%) compared to platinum-resistant patients (16.7%).^531^ The study also reported a median PFS of 8.1 months and noted that 89.5% of participants experienced at least one treatment-related adverse event, with 23.7% experiencing a grade 3 or higher event. Interestingly, responses to the combination therapy occurred across PD-L1 expression levels, suggesting that nivolumab with bevacizumab exhibits activity in relapsed ovarian cancer, particularly in the platinum-sensitive subgroup, and highlighting the need for alternative strategies in platinum-resistant cases.^531^ Similarly, the phase III IMpower150 study (NCT02366143) assessed atezolizumab plus bevacizumab and chemotherapy in non-squamous NSCLC patients, revealing significantly better response rates and survival outcomes compared to control groups, independent of PD-L1 expression and effector T cell status.^532^

Apart from anti-VEGF antibody such as bevacizumab, VEGFR tyrosine kinase inhibitors (TKIs) blocking intracellular transduction of VEGF signaling are also widely used in clinical practice, including axitinib, sorafenib, vatalanib, apatinib, and sunitinib.^533^ In the phase III JAVELIN Renal 101 trial, the combination of avelumab (anti-PD-1 antibody) and axitinib was compared to standard-of-care sunitinib in previously untreated patients with advanced renal-cell carcinoma.^534^ For PD-L1-positive patients, median PFS was significantly longer for the combination therapy (13.8 months) compared to sunitinib (7.2 months), with an HR for disease progression or death at 0.61.^534^ In the overall patient population, the median PFS was 13.8 months with the combination therapy versus 8.4 months with sunitinib, indicating a benefit across a broader group. The ORR among PD-L1-positive patients was 55.2% with the combination therapy compared to 25.5% with sunitinib.^534^ These findings demonstrate that avelumab plus axitinib significantly improves PFS over sunitinib in first-line treatment for advanced renal-cell carcinoma, presenting a potent treatment option for this patient population.^534,535^ Additionally, Xu et al. reported a phase I study (NCT02942329) combining SHR-1210 (anti-PD-1 antibody) with apatinib in various cancers, noting particularly favorable results in hepatocellular carcinoma patients.^536^ These studies collectively underscore the potential of combining anti-PD-L1 with anti-angiogenesis agents to enhance therapeutic efficacy across multiple cancer types.

### Agents targeting EGF/EGFR and other growth factors

#### Anti-EGF/EGFR therapy

The discovery and subsequent elucidation of the EGFR signaling pathway represent a cornerstone in our understanding of cellular proliferation and oncogenesis. Initially identified in the 1960s through the pioneering work of Cohen, who discovered the EGF and its role in stimulating epithelial cell proliferation,^537^ and later Carpenter, who identified the specific receptor for EGF, EGFR has been established as a crucial receptor tyrosine kinase (RTK) in mediating cell growth and survival signals.^538^ As part of the ErbB family of RTKs, which includes HER2, HER3, and HER4, EGFR plays a pivotal role in organ development and tissue repair under physiological conditions.^539^ However, its deregulation, through mutations or overexpression, activates a cascade of pro-survival and anti-apoptotic signaling pathways leading to tumorigenesis in various cancers, including NSCLC, colorectal cancer, and glioblastoma.^540–544^ Hereto, targeting the EGFR pathway has emerged as a cornerstone in the treatment of certain cancers, reflecting a strategic shift towards precision oncology. The therapeutic arsenal against EGFR-driven malignancies includes small molecule TKIs and monoclonal antibodies, which respectively target the receptor intracellular kinase domain to prevent activation and its extracellular domain to block ligand binding and receptor dimerization (Table 19).^545–547^ Despite the clinical benefits of these agents, the challenge of acquired resistance has spurred ongoing research into novel therapeutic strategies.^548–551^ These include the development of combination therapies, as well as innovative approaches like peptides, nanobodies, and therapeutic vaccines aimed at directly inhibiting the EGFR or its ligands.^552–556^ Such advancements hold promise for overcoming resistance mechanisms and enhancing treatment outcomes, underscoring the dynamic evolution of cancer therapy in the era of molecular targeting.

**Table 19:** EGFR-TKIs targeting common EGFR mutations

Generation	Target	Agent	Type of ATP-competitive inhibitor	Status
First generation	Del19/L858R	Gefitinib	Reversible	FDA approval
		Erlotinib	Reversible	FDA approval
		Icotinib	Reversible	NMPA approval
Second generation	Del19/L858R	Afatinib	Irreversible	FDA approval
		Dacomitinib	Irreversible	FDA approval
Third generation	T790M	Osimertinib	Irreversible	FDA approval
		Almonertinib	Irreversible	NMPA approval
		Lazertinib	Irreversible	Phase III (NCT04248829, NCT04487080, NCT05388669, NCT04988295)
		BPI-7711	Irreversible	Phase III (NCT03866499)
		SH-1028	Irreversible	Phase III (NCT06080776, NCT04239833)
		ASK120067	Irreversible	Phase III (NCT04143607)
	ex20ins	Mobocertinib	Irreversible	FDA approval
		Sunvozertinib	Irreversible	NMPA approval
		Furmonertinib	Irreversible	NMPA approval
		Poziotinib	Irreversible	Phase III (NCT05378763)
		CLN-081	Irreversible	Phase III (NCT05973773)

Note: *FDA* Food and Drug Administration, *NMPA* National Medical Products Administration

The exploration and development of anti-EGFR therapies have significantly evolved, offering new avenues for cancer treatment through a deep understanding of EGFR biochemistry and mechanisms underlying therapeutic resistance.^557^ Conventional EGFR inhibitors, such as TKIs and monoclonal antibodies, have been foundational in treating EGFR-driven tumors, transforming the management of cancers, especially NSCLC and colorectal cancer.^558–560^ The first-generation TKIs, exemplified by gefitinib, targeted EGFR with a degree of success limited to patients with specific EGFR mutations.^561^ This limitation, coupled with the emergence of resistance, led to the development of subsequent generations of TKIs aimed at offering more durable control of cancer progression by targeting additional resistance mechanisms, including the T790M mutation.^562^ However, despite these advancements, resistance remains a significant challenge, prompting research into fourth-generation TKIs and alternative strategies such as blocking the EGFR ligand, EGF, directly with promising early results from therapeutic vaccines, peptides, and single-domain antibodies.^563–565^

Besides TKIs, monoclonal antibodies against EGFR, such as cetuximab, panitumumab, amivantamab, and necitumumab, have also been approved for treating various cancers, often in combination with chemotherapy.^566–571^ Yet, the efficacy of these antibodies is hampered by resistance mechanisms similar to those affecting TKIs, including mutations in the EGFR extracellular domain and alterations in downstream signaling pathways.^572,573^ Strategies are being explored to address the issue of acquired resistance to existing anti-EGFR monoclonal antibodies, including the combination of antibodies that target distinct, non-overlapping regions of EGFR.^574^ Furthermore, the combination of anti-EGFR therapies with other treatment strategies, such as ICB and chemotherapy, represents a promising strategy to overcome resistance and improve patient outcomes.^575–579^ For instance, in a phase II trial involving 33 participants with recurrent or metastatic head and neck squamous cell carcinoma, the combination of pembrolizumab and cetuximab achieved the 45% ORR at 6 months, with the most common serious adverse event being oral mucositis.^575^ The challenge lies in the intricate nature of cancer cell survival mechanisms, requiring a multifaceted approach that includes the simultaneous targeting of EGFR and other critical pathways involved in tumor growth and progression. As the landscape of anti-EGFR therapy continues to expand, future research will likely focus on optimizing combination treatments, developing novel inhibitors that can bypass or prevent resistance, and refining patient selection criteria to maximize therapeutic efficacy and durability.

#### Anti-HER2 agents

Apart from EGFR, human epithelial growth factor receptor 2 (HER2) also belongs to the EGF receptor tyrosine kinase family.^580^ HER2 plays a pivotal role in the development, progression, and prognosis of various cancers due to its gene amplification or receptor overexpression.^581^ Notably, HER2 positivity is observed in a significant percentage of breast and gastric cancers, making HER2 a key target for diagnosis and treatment.^582–584^ The absence of a natural ligand for HER2 distinguishes it from other family members, with its activation primarily through dimerization with other receptors.^585^ HER2-targeted therapies, including trastuzumab and pertuzumab monoclonal antibodies, TKIs like lapatinib and afatinib, and the antibody-drug conjugate including T-DM1 and DS8201, have significantly advanced the treatment of HER2-positive cancers, particularly breast cancer.^586–593^ These therapies work by inhibiting HER2 dimerization, blocking downstream signaling pathways, inducing antibody-dependent cellular cytotoxicity, and delivering cytotoxic agents directly to cancer cells.^594–596^ Despite the success, resistance to treatments such as trastuzumab remains a challenge, prompting ongoing research into combination therapies and the development of novel anti-HER2 agents to enhance treatment efficacy and overcome resistance.^597,598^

#### Anti-FGFR therapy

Additionally, the fibroblast growth factor receptor (FGFR) pathway plays a significant role in cellular functions such as proliferation, differentiation, and survival, which are critical in both development and cancer progression.^599^ Aberrations in FGFR signaling, including gene amplifications, mutations, and alterations in ligand specificity through alternative splicing, contribute to oncogenesis and cancer progression across various tumors.^600^ Anti-FGFR therapies have emerged as promising strategies in cancer treatment. These therapies encompass a range of approaches, including TKIs like pemigatinib, futibatinib, erdafitinib and infigratinib, which have shown efficacy in cancers with FGFR genetic alterations, and monoclonal antibodies targeting FGFRs or their ligands to block aberrant signaling pathways (Table 20).^601–605^ For example, in a phase III trial, erdafitinib significantly improved OS compared to chemotherapy in patients with FGFR-altered metastatic urothelial carcinoma post-ICB, achieving a median survival of 12.1 vs. 7.8 months (HR: 0.64; *P* = 0.005).^606^ Despite the potential, challenges such as drug resistance and the intricate role of FGFR in normal physiology necessitate further research to optimize anti-FGFR therapies in cancer.^607^

**Table 20:** Clinical trials of the combination therapy of FGFR blockade and immune checkpoint inhibitor

FGFR inhibitor	NCT number	Cancer types	Combination partners	Phase	Status
Pemigatinib	NCT05913661	Intrahepatic Cholangiocarcinoma	PD-1 Inhibitors	II	Recruiting
	NCT05004974	NSCLC	Sintilimab	II	Recruiting
	NCT04949191	Solid Tumors	Pembrolizumab	II	Active, not recruiting
	NCT04003610	Urothelial Carcinoma	Pembrolizumab	II	Terminated
	NCT02393248	Solid Tumors	Pembrolizumab	I/II	Terminated
Futibatinib	NCT05945823	Solid Tumors	Pembrolizumab and Chemotherapy	II	Recruiting
	NCT05036681	Microsatellite Stable Endometrial Carcinoma	Pembrolizumab	II	Recruiting
	NCT04828486	Hepatocellular Carcinoma	Pembrolizumab	II	Recruiting
	NCT04601857	Urothelial Cancer	Pembrolizumab	II	Recruiting
Erdafitinib	NCT05564416	Bladder Cancer	Atezolizumab	II	Withdrawn
Infigratinib	NCT05510427	Cholangiocarcinoma	Atezolizumab and Bevacizumab	I	Withdrawn
HMPL-453	NCT05173142	Solid Tumors	Gemcitabine, Cisplatin, Toripalimab, and Docetaxel	I/II	Recruiting
Bemarituzumab	NCT05322577	Gastric or Gastroesophageal Junction Cancer	CAPOX/SOX and Nivolumab	I	Recruiting
	NCT05267470	Squamous-Cell NSCLC	Pembrolizumab, Carboplatin and Paclitaxel/Nab-paclitaxel	I	Active, not recruiting
	NCT05111626	Gastric or Gastroesophageal Junction Cancer	Chemotherapy (mFOLFOX6 or CAPOX) and Nivolumab	III	Recruiting

Note: *NSCLC* non-small cell lung cancer

#### Strategies targeting HGF/c-MET signaling

The HGF/c-MET signaling pathway, fundamental in cell growth and organ regeneration, has been implicated in cancer progression and metastasis due to its role in cellular proliferation, survival, and migration.^608^ HGF, produced by stromal cells, activates the c-MET receptor tyrosine kinase, leading to various downstream effects including cell scattering, invasion, and angiogenesis.^609–611^ Alterations in this pathway, such as MET gene amplification, overexpression, or activating mutations, have been identified in several cancers, contributing to tumor growth, angiogenesis, and resistance to therapies.^612–615^ Consequently, targeting the HGF/c-MET axis has emerged as a therapeutic strategy, with development of inhibitors like crizotinib and cabozantinib showing efficacy in certain cancers by blocking MET kinase activity.^616–619^ In a phase II trial (NCT01945021), crizotinib (inhibitor of ALK, ROS1, and MET) showed significant efficacy and durable responses in patients with ROS1-positive advanced NSCLC, achieving an ORR of 71.7% and median PFS of 15.9 months, with a safety profile consistent with previous studies.^620^ Besides, in the phase III trial CELESTIAL, cabozantinib (inhibitor of VEGFR, MET, and AXL) extended median OS to 10.2 months compared to 8.0 months with placebo (HR: 0.76; *P* = 0.005) and improved median PFS to 5.2 months versus 1.9 months (HR: 0.44; *P* < 0.001) in previously treated advanced hepatocellular carcinoma patients.^621^ Also, in the phase III trial CheckMate-9ER, nivolumab plus cabozantinib continued to show superior efficacy over sunitinib in first-line treatment of advanced renal cell carcinoma, with the median OS of 37.7 months compared to 34.3 months (HR: 0.70, *P* = 0.0043) and the median PFS of 16.6 months versus 8.3 months (HR: 0.56, *P* < 0.0001).^622^ Additionally, monoclonal antibodies targeting HGF/c-MET signaling are being investigated to inhibit ligand-receptor interactions, further exploring its potential as a target for cancer therapy.^623,624^ Notably, the HGF/c-MET signaling pathway is frequently hijacked by cancer cells to develop resistance to chemotherapy, radiotherapy, and targeted therapies, such as gefitinib and sotorasib.^625–628^ Consequently, targeting the HGF/c-MET signaling axis represents a promising strategy in cancer treatment, particularly when used combined with other therapeutic modalities.

#### Harnessing other growth factors

Besides, other protumor growth factor pathways such as the PDGF/PDGFR signaling, crucial for tumor cell proliferation, invasion, metastasis, and angiogenesis, present potential anti-cancer targets, with emerging therapies showing effectiveness yet facing challenges with efficacy and toxicity in clinical trials.^629,630^ Conversely, the hematopoietic growth factor GM-CSF acts as a tumor suppressor in most cases by eliciting immune responses against tumors.^631^ It enhances antitumor immunity primarily through the activation and maturation of DCs and macrophages, Th9 cell responses, and eventually inducing T cell cytotoxicity against tumors.^632–635^ The therapeutic applications of GM-CSF extend from counteracting neutropenia in cancer patients, to serving as an adjuvant in cancer vaccines where it boosts antigen presentation and T cell activation, thereby improving vaccine efficacy.^636^ Furthermore, GM-CSF-expressing oncolytic virus therapies and GM-CSF-secreting tumor cell vaccines have shown promise in inducing potent antitumor immune responses.^637–640^ However, GM-CSF can also promote tumor progression by enhancing MDSCs and TAMs, indicating the complexity of its effects in the TME.^641,642^ This dual nature of GM-CSF necessitates a careful balance in its application, which underscores the importance of dosage, administration route, and combination with other therapeutic strategies to maximize its antitumor potential while minimizing protumor effects.^643^

In a pioneering phase I clinical trial, an innovative autologous GM-CSF-secreting breast cancer vaccine was administered to patients with both metastatic (*n* = 12) and stage II-III breast cancer (*n* = 7), showcasing limited toxicity alongside variable efficacy.^644^ Notably, among those with metastatic disease, eight developed disease progression within two months, whereas one remarkable case exhibited no evidence of disease for an extended period of 13 years; patients with stage II-III breast cancer reported a median survival time of 6.24 years following vaccination.^644^ Furthermore, the phase II trial (ChiCTR1900026175) assessed the efficacy and safety of the PRaG regimen (a combination of radiotherapy, anti-PD-1, and GM-CSF) in patients facing metastatic cancer resistant to conventional treatments.^645^ This trial, with a median follow-up period of 16.4 months across 54 participants, revealed an ORR of 16.7%, a DCR of 46.3%, and a median PFS of 4.0 months alongside an OS of 10.5 months^645^. Lastly, the phase II trial (NCT01767194) explored the I/T/DIN/GM-CSF regimen (irinotecan, temozolomide, dintuximab, and GM-CSF) for patients with relapsed/refractory neuroblastoma, confirming its significant antitumor efficacy.^646^ Out of 53 patients, 22 (41.5%) achieved objective responses, with another 22 maintaining stable disease.^646^ This regimen showed a one-year PFS rate of 67.9% and an OS rate of 84.9% with a tolerable safety profile, prompting further research into its application in frontline treatments and the exploration of predictive biomarkers.^646^

## Clinical progress and future direction

The clinical progress of cytokine and chemokine-targeted therapies has been marked by both challenges and significant achievements. The journey from preclinical research to clinical application has illuminated the nuanced role these molecules play in cancer biology, offering novel therapeutic avenues that extend beyond traditional treatment modalities.

The approval of cytokine therapies such as IFN-α and IL-2 for the treatment of certain cancers has been a testament to the clinical potential of targeting cytokine pathways. IFN-α, utilized for its immunomodulatory and anti-proliferative properties, has been approved for melanoma, follicular lymphoma, and other malignancies. IL-2, known for its capacity to boost T-cell responses, has been approved for metastatic renal cell carcinoma and metastatic melanoma, demonstrating the feasibility of enhancing the immune system to fight cancer. These approvals were based on clinical trials that highlighted the efficacy of these cytokines in improving patient outcomes, albeit with the recognition of their limitations, including severe side effects and the need for high-dose administration. Besides, a significant area of clinical research has focused on combining cytokine therapies with ICB to overcome resistance mechanisms that limit the efficacy of ICB alone. Numerous trials have evaluated the combination of ICB with cytokines like IFN-α and IL-12, revealing that such combinations can synergistically enhance antitumor immunity. For example, clinical trials combining IFN-α with pembrolizumab in melanoma patients have demonstrated improved response rates compared to pembrolizumab monotherapy, indicating the potential of IFN-α to augment the immune system to recognize and destroy tumor cells.

Moreover, the clinical development of monoclonal antibodies and receptor inhibitors targeting protumor cytokines such as VEGF, IL-6, and TGF-β represents another milestone in cancer therapy. Bevacizumab, an anti-VEGF antibody, has been widely incorporated into treatment regimens for colorectal cancer, NSCLC, hepatocellular carcinoma, and glioblastoma. Similarly, inhibitors targeting the IL-6 pathway, like tocilizumab, have entered clinical trials to evaluate their potential in mitigating cancer-related inflammation and cachexia, showcasing the therapeutic versatility of targeting cytokine pathways. Also, anti-TGF-β/PD-L1 bifunctional antibodies such as M7824 have demonstrated its potential in treating various advanced solid tumors, including NSCLC and cervical cancer. It has been acclaimed as a “next-generation” anti-PD-L1 agent, exemplified by a phase I clinical trial that reported an outstanding response rate of over 85% in patients with PD-L1^high^ NSCLC.

Despite these advances, the clinical application of cytokine and chemokine-targeted therapies is not without challenges. The adverse effects associated with cytokine therapies, such as the capillary leak syndrome seen with high-dose IL-2 treatment, have necessitated the development of strategies to mitigate toxicity while preserving efficacy. Moreover, the heterogeneity of tumors and the complexity of the TME mean that not all patients respond equally to these treatments, underscoring the need for biomarkers to predict response and guide therapy selection. Take the M7824 as an example, despite the promising early results in phase I clinical trials, subsequent larger-scale clinical trials of anti-TGF-β/PD-L1 therapies encountered unforeseen challenges. M7824 did not meet the primary endpoint event in multiple phase II/III clinical trials including biliary tract cancer and NSCLC. The underlying reasons for these setbacks remain unclear, it is generally believed that optimizing patient selection is crucial for the successful clinical translation.

Looking ahead, the clinical development of cytokine and chemokine-targeted therapies is poised to benefit from advancements in precision medicine, biomarker research, and drug delivery systems. The ongoing integration of these therapies with other treatment modalities, coupled with a deeper understanding of their mechanisms of action, promises to expand their therapeutic potential and refine their clinical application, ultimately improving outcomes for patients with cancer.

## Conclusion and perspective

**Fig. 9:**
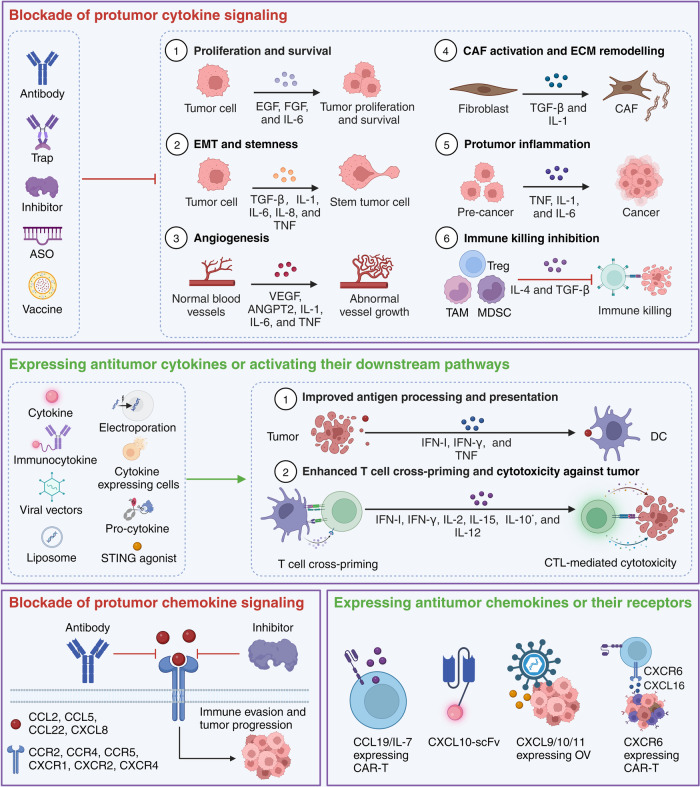
Cytokine- and chemokine-targeted strategies in cancer therapy. The top panel illustrates various methods of blocking protumor cytokine signaling pathways, including antibody, trap, inhibitor, antisense oligonucleotide (ASO), and vaccine. The middle panel depicts strategies for expressing antitumor cytokines or activating their downstream pathways, including direct cytokine or modified cytokine administration, electroporation, immunocytokines, cytokine-expressing cells, viral vectors, liposome delivery, and STING agonists. Notably, IL-10 generally suppresses immune response, but some studies suggest that it promotes the activation of tumor-resident CD8^+^ T cells. Therefore, IL-10 administration is used to improve immunotherapy effectiveness in some clinical trials. The bottom left panel highlights the blockade of protumor chemokine signaling using antibodies and inhibitors to disrupt mechanisms contributing to immune evasion and tumor progression. The bottom right panel presents approaches for expressing antitumor chemokines or their receptors to stimulate an immune response, featuring engineered T cells expressing specific chemokine receptors and oncolytic viruses (OVs) designed to deliver chemokine genes directly into the tumor microenvironment. Adapted from “The Tumor Microenvironment: Overview of Cancer-Associated Changes”, by BioRender.com (2024). Retrieved from https://app.biorender.com/biorender-templates

The past few decades have witnessed significant advances in understanding the complex interplay between cytokines, chemokines, and their signaling pathways in the context of cancer biology. These insights have paved the way for innovative therapeutic strategies targeting cytokine and chemokine signaling pathways, offering new hope for patients with cancer. The development of cytokine-based therapies, including both antagonists and agonists, has underscored the dual nature of these molecules in cancer, where they can act as both promoters and suppressors of tumorigenesis depending on the context. This duality presents both challenges and opportunities for therapeutic intervention, necessitating a refined approach to harnessing their potential for cancer therapy. The emergence of targeted therapies against specific cytokines, such as IFN-I, IL-2, and IL-12, has demonstrated the feasibility of modulating the immune system to combat cancer. Similarly, the blockade of protumor cytokines, including TGF-β, VEGF, and IL-6, using antibodies and small-molecule inhibitors, has shown promise in inhibiting tumor growth, metastasis, and angiogenesis (Fig. 9) (Table 21). These therapies not only direct their effects on the tumor cells but also remodel the TME to enhance antitumor immunity. Furthermore, the advent of combination strategies, particularly the synergy between cytokine blockade and ICB, has opened new avenues for overcoming resistance to conventional immunotherapies and improving patient outcomes.

**Table 21:** Cytokines targeted for cancer treatment

Cytokine	Source	Main generation mechanisms	Main biological activities harnessed by cancer therapy
IFN-Is	Immune cells, tumors cells, endothelial cells, CAFs	TLR4-MyD88 pathway, cGAS/STING pathway, and TLR3-TICAM1 pathway	Inducing tumor cell apoptosis; Promoting the maturation and antigen presentation of DCs; Enhancing NK activation (IFN-I therapy or agonist).
IFN-γ	NK cells and T cells	Both receptor- and cytokine-dependent mechanisms	Promoting the antigen presentation of macrophages and DCs; Enhancing the activation of T cells and NK cells; Inhibiting Treg functions; Hampering Th2 and Th17 response (IFN-γ therapy).
IL-1	IL-1α: immune cells and non-immune cells; IL-1β: immune cells	IL-1α: constitutively expressed and upregulated by inflammatory stimuli and oxidative stress; IL-1β: induced by inflammatory stimuli	Increasing the accumulation of TAMs and MDSCs (counteracted by IL-1 inhibitors).
IL-2	T cells	TCR stimulation	Promoting the function and activity of T cells and NK cells; Improving memory T cell development (engineered IL-2 proteins).
IL-6	Immune cells and non-immune cells	Cancer-related inflammation	Promoting cancer cell proliferation, survival, metastasis, angiogenesis, and immune evasion (inhibitors of IL-6 and downstream signaling).
IL-10	Immune cells, tumor cells, epithelial cells	PRR stimulation	Generally, IL-10 suppresses immune response, but some studies suggest that it promotes the activation of tumor-resident CD8^+^ T cells (IL-10 therapy).
IL-12	DCs, macrophages, and B cells	PRR stimulation, IFN-dependent pathway	Enhancing the function and activity of T cells and NK cells; Promoting Th1 response; Reprograming immunosuppressive cells, such as MDSCs and TAMs (localized IL-12 delivery).
IL-15	Mainly expressed by myeloid cells	Regulated at multiple levels: transcription (IRF-E and NF-κB), post-transcription, and trans-presentation (IL-15Rα).	Promoting the function and activity of T cells and NK cells (engineered IL-15 proteins).
TNF	Immune cells and non-immune cells	Cancer-related inflammation	Promoting cancer cell proliferation, survival, metastasis, angiogenesis, and immune evasion (TNF blockade).
VEGF	Immune cells and non-immune cells	Hypoxia	Supporting tumor angiogenesis, growth, and metastasis; Undermining the functions of effector cells and DCs; Increasing the accumulation of immunosuppressive cells (Blocked by anti-angiogenesis agents)
TGF-β	Tumor cells and stromal cells	Latent TGF-β complex is activated by integrins, acids-bases, ROS, proteases	Promoting tumor epithelial-mesenchymal transition, metastasis, treatment resistance, and matrix remodeling; Inducing the differentiation of Tregs, M2-like macrophages, MDSCs; Hampering the functions of NK cells, T cells, and DCs (TGF-β and PD-L1 dual blockade).

Note: *CAF* cancer-associated fibroblast, *DC* dendritic cell, *IRF-E* interferon regulatory factor element, *MDSC* myeloid-derived suppressor cell, *NK cell* natural killer cell, *PRR* pattern recognition receptor, *ROS* reactive oxygen species, *TAM* tumor-associated macrophage, *Treg* regulatory T cell

Notably, the pleiotropic nature of cytokines and their context-dependent roles in cancer and immunity necessitate a deeper understanding of the TME and the dynamic interactions between different cell types. This complexity underscores the need for precision medicine approaches that consider individual patient characteristics, including the genetic and molecular profiles of tumors, to tailor therapies for optimal efficacy. Moreover, the development of predictive biomarkers to identify patients who are most likely to benefit from specific cytokine-targeted therapies is crucial for advancing personalized cancer treatment. In the future, continued research into the biology of cytokines, along with technological advancements in drug delivery and molecular engineering, holds the promise of developing more effective and less toxic therapeutic options. The integration of cytokine-based therapies with other treatment modalities, such as targeted therapies, chemotherapy, and radiotherapy, offers a comprehensive approach to cancer management. Furthermore, the exploration of novel targets and mechanisms of action, including the modulation of the immune system and the TME, will likely yield additional therapeutic candidates.

In conclusion, targeting cytokine and chemokine signaling pathways represents a frontier in cancer therapy, offering the potential to significantly improve patient outcomes. The successes achieved so far provide a strong foundation for future research and clinical development. By leveraging our growing understanding of cytokine biology, coupled with advancements in biotechnology and precision medicine, we can look forward to more effective, personalized therapies to fight cancer. The translation from the bench to the bedside is fraught with challenges, but the promise of cytokine- and chemokine-targeted therapies in revolutionizing cancer treatment is undeniably within reach.
